# Citizen science for monitoring seasonal-scale beach erosion and behaviour with aerial drones

**DOI:** 10.1038/s41598-021-83477-6

**Published:** 2021-02-16

**Authors:** Nicolas Pucino, David M. Kennedy, Rafael C. Carvalho, Blake Allan, Daniel Ierodiaconou

**Affiliations:** 1 School of Life and Environmental Sciences, Deakin University, Warrnambool, Australia; 2 School of Geography, The University of Melbourne, Melbourne, Australia

**Keywords:** Geomorphology, Natural hazards, Climate-change mitigation

## Abstract

Sandy beaches are highly dynamic systems which provide natural protection from the impact of waves to coastal communities. With coastal erosion hazards predicted to increase globally, data to inform decision making on erosion mitigation and adaptation strategies is becoming critical. However, multi-temporal topographic data over wide geographical areas is expensive and time consuming and often requires highly trained professionals. In this study we demonstrate a novel approach combining citizen science with low-cost unmanned aerial vehicles that reliably produces survey-grade morphological data able to model sediment dynamics from event to annual scales. The high-energy wave-dominated coast of south-eastern Australia, in Victoria, is used as a field laboratory to test the reliability of our protocol and develop a set of indices to study multi-scale erosional dynamics. We found that citizen scientists provide unbiased data as accurate as professional researchers. We then observed that open-ocean beaches mobilise three times as much sediment as embayed beaches and distinguished between slowed and accelerated erosional modes. The data was also able to assess the efficiency of sand nourishment for shore protection. Our citizen science protocol provides high quality monitoring capabilities, which although subject to important legislative preconditions, it is applicable in other parts of the world and transferable to other landscape systems where the understanding of sediment dynamics is critical for management of natural or anthropogenic processes.

## Introduction

The coastal zone accommodates 40% of today’s population^1,2^, with densities much greater than non-coastal land especially in low income countries^3^. Sandy beaches are extremely dynamic morphological sub-systems of the coastal zone, offering, amongst others, coastal protection and erosion control eco-services^4,5^. However, global studies have reported that one-quarter of the world’s sandy beaches are eroding at rates exceeding 0.5 m/year^6^, contributing to a global coastal land loss of 20,000 km^2^ in the last 35 years^7^, posing an increasing threat to coastal populations and economies. Beach erosion is not uniformly distributed along the coast, but concentrated on discrete areas^6,7^ often referred as erosional hotspots^8,9^. These hotspots are spatiotemporally variable ranging from days to decades and from hundreds to thousands of meters^10^. As a consequence, erosion mitigation strategies (beach nourishment, rockwalls, sand fencing) tend to be highly localized^11^. In these areas, the measurement of high-temporal volumetric variations is critical for discerning short-term beach behaviour and recovery from erosional events^12^. This allows coastal planners to target intervention measures and evaluate their efficiency in protecting backshore assets from current and projected increase of erosion hazard^13–15^.

Nowadays, beach topographic data is obtainable using a variety of ground-based (graded rods^16^, surveyor-grade global positioning systems or total stations^17^, terrestrial LiDAR^18^) or aerial-based (airborne LiDAR^19^, traditional photogrammetry^20,21^, unmanned aerial vehicles^22,23^) approaches. While ground-based surveying methods are labour intensive and of limited spatial coverage, airborne LiDAR or traditional photogrammetric approaches are cost-prohibitive for monitoring purposes, especially in developing countries.

In the last decade unmanned aerial vehicles combined with structure-from-motion multi-view stereo algorithms (UAV-SfM hereinafter) have emerged in environmental research^24–26^ as the best compromise in terms of costs, precision, reproducibility and simplicity, particularly for repetitive beach topographic surveys^27–29^. In coastal areas, multi-rotor UAVs are frequently used^30^ and their flight time is typically between 20 and 40 min per battery, which corresponds to a flight coverage of 5–30 × 10^3^ m^2^, depending on flight altitude^24^. As a consequence, time and budget constraints have so far restricted UAV-SfM surveys to professional researchers or commercial operators at a few representative sites, with limited revisit times^27,31–35^.

However, recent technological advances in low-cost UAVs and automated flight and positioning solutions, coupled with regulatory changes, provide new opportunities for implementing citizen science to expand the scale of monitoring programs^36,37^. Citizen science is the process of creating knowledge by engaging non-professional volunteers in scientific research^36^. Citizen scientists have allowed large-scale scientific experiments to make substantial contributions to science for hundreds of years^38^. Yet, errors and bias in large and longitudinal citizens science datasets are often poorly understood^39^. This uncertainty, being difficult to quantify, can compromise data quality or limit the integration of multiple datasets into a single coherent analysis. As a result scepticism exists among professional scientists about the quality of citizen scientists’ data^40,41^.

In this study, we propose a novel and cost-effective approach to monitor sandy beaches sediment dynamics at a spatiotemporal resolution previously unachievable, through citizen science with low-cost multi-rotors UAVs. We test our method on the high-energy temperate coast of Victoria, Australia, where more than 100 citizen scientists collected 83 aerial datasets in ten previously identified erosional hotspots over a 1.3 year period, approximately every 6 weeks. With this unique dataset, we first evaluate citizen scientists' data quality and bias, then we quantify and compare short-term volumetric and profile dynamics on both open-ocean and embayed beaches. We propose a novel set of indices that capture multi-scale landform dynamics, using topography timeseries alone. This allows us to evaluate beach nourishment efficiency in protecting backshore infrastructure. Lastly, we discuss the potential and limitations that our approach has not only for coastal management but also in other scientific disciplines.

## Results

### Citizen scientist’s data accuracy and bias

**Figure 1:**
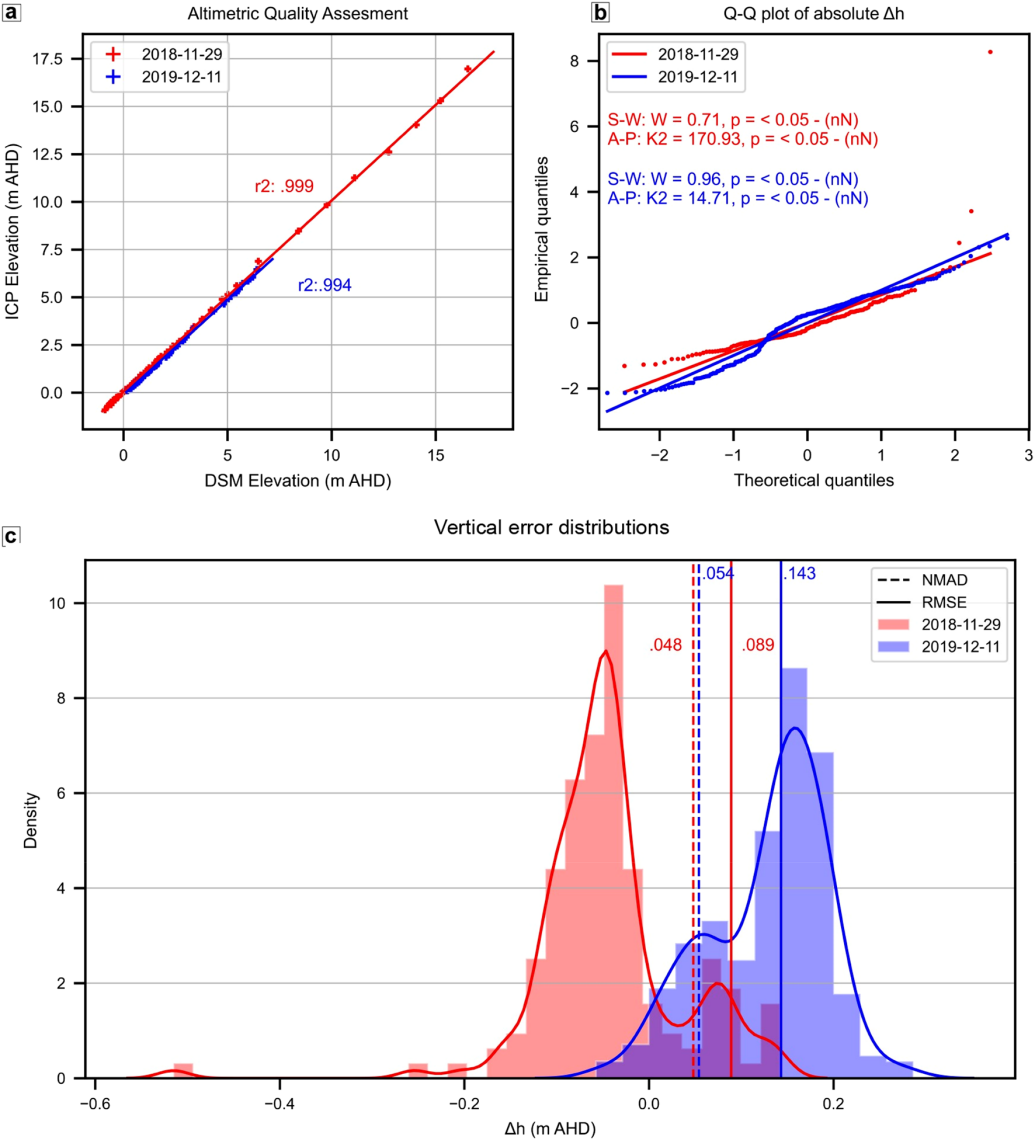
Independent checkpoints (ICP) analysis (n = 464) shows that citizen scientists' digital surface models (DSM) vertical accuracy is 0.048 m using smart ground control points in the operational scenario (2018-11-29, in red, always implemented) or 0.054 m in the worst case scenario (2019-12-11, in blue) of post-survey manual georeferencing, demonstrating that citizen scientists acquire surveyor grade data of the same quality as academic researchers. (**a**) Comparison between modelled elevation from the DSM and ICP elevations showing very good linear agreement in both surveys. (**b**) Q–Q plots visually rejecting normality assumption of the absolute error (Δ*h*) in both surveys, with Shapiro–Wilk (S–W) and D’Agostino–Pearson (A–P) normality tests reinforcing the non-normality (nN) visual observation at the 0.05 significance level. (**c**) Density histograms showing the skewing due to heavy tails in both surveys and the robustness of nmad to outliers. The root mean squared error (rmse) is also reported for comparison purposes.

Accuracy and bias are objective task-independent metrics of data quality^42^. For an independent and realistic vertical accuracy assessment of the digital surface models (DSM), the error metrics should be calculated with independent checkpoints that haven’t been used during the digital photogrammetric procedure (Supplementary Method “Citizen Scientists, UAV surveys and photogrammetric details”) and are distributed across the landscape, along representative transects or landform elements^27,43^. Two benchmark surveys were performed in Warrnambool to evaluate the DSM vertical accuracy resulting from the citizen science protocol under operational (2018-11-29) and worst-case (2019-12-11) scenarios (Supplementary Method “Independent checkpoint surveys”). The checkpoints in both surveys show a very good linear match of modelled elevation (both R^2^ above 0.99) with slightly larger deviations observed at higher elevations (Fig. 1a).

The Q–Q plots and statistical tests indicate non-normal distribution of absolute errors (Δ*h,* Fig. 1b), which confirms that the normalised median absolute deviation (nmad) is the most appropriate robust estimator for vertical accuracy assessment of photogrammetric datasets^44^. The nmad values are 0.048 and 0.054 m AHD (Australian height datum) for the operational and worst scenarios respectively. The mean errors indicate that the 2018 checkpoints survey slightly underestimated the height values (− 0.044 m AHD), the 2019 checkpoints survey overestimated (0.128 m AHD) it, which is observable from the two error distributions (Fig. 1c). Both surveys show good precisions (standard deviation) of 0.077 m AHD and 0.063 m AHD respectively. Supplementary Table S3 also reports root mean squared error (rmse) and mean absolute error for comparison purposes. Casella et al. ^45^ recently demonstrated that approximately 0.05 m rmse is consistently found under different surveying set-ups (varying cameras and flight altitudes), which corroborates a median rmse of 0.059 m found in the relevant literature (Supplementary Table S2). The authors state this systematic error could be due to the vertical sinking of the surveying pole on various sand types, therefore, 0.05 m rmse is a typical error in sandy beaches UAV-SfM studies.

As our operational scenario rmse (0.089 m AHD) is about 0.03 m higher than the literature median, we further explored systematic errors by mapping the spatial variability of checkpoints errors and smart ground control points (GCPs) (Supplementary Figs. S2, S3), finding that errors were generally higher with elevation, especially within foredune vegetation. We mitigated this systematic error by removing vegetation and applying specific limits of detection thresholds, which is a form of split data test^46^ used to obtain the expected DSMs vertical errors by computing the elevation difference between pre- and post- survey pairs over known stable areas (i.e. calibration areas^22^).

**Figure 2:**
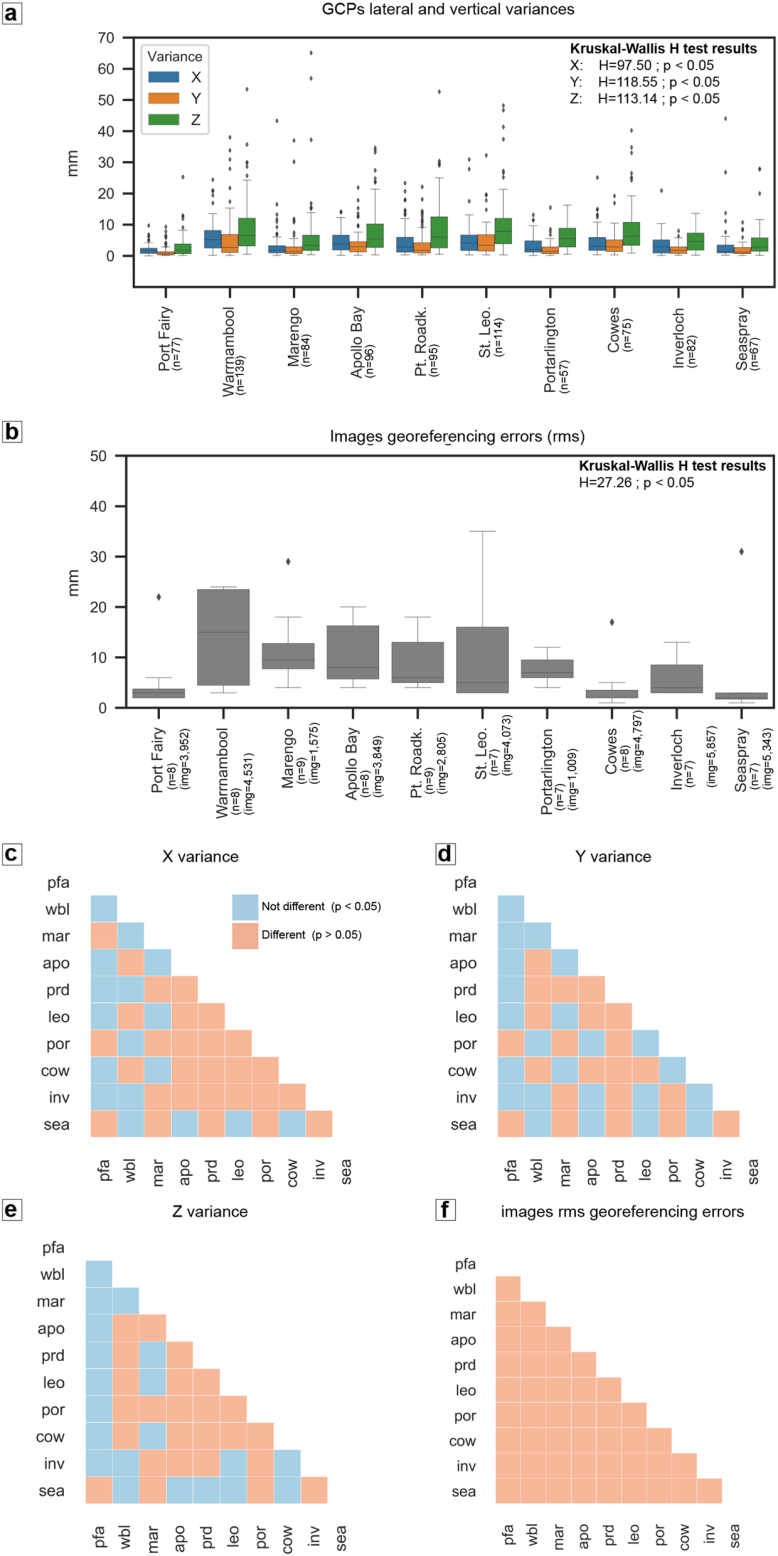
Bias analysis for ground control points (GCPs) characteristics across all surveys. (**a**): horizontal (X and Y) and vertical (Z) GCPs coordinate variances obtained while recording their position during (*n*) surveys in each location; (**b**) Images georeferencing errors computed with the total images (*img*) in all locations; (**c**–**f**) pairwise significant (red) and non-significant (blue) differences across locations, resulting from the post hoc Dunn’s test^48^ (*p* = 0.05, adjusted using a step-down method with Bonferroni adjustments). Note that six photogrammetric or GCPs reports were not available for this analysis. Location codes: pfa = Port Fairy; wbl = Warrnambool; mar = Marengo; apo = Apollo Bay; prd = Pt. Roadk.; leo = St. Leo.; por = Portarlington; cow = Cowes; inv = Inverloch; sea = Seaspray.

Bias in citizen science projects can be introduced by allowing individuals flexibility in how, when and where to collect data^47^. We reduced the risk of bias prior UAV operations by (1) assigning groups to fixed locations, (2) targeting low-tide for survey, (3) providing standardised protocols and training on simple to use highly automated equipment and (4) assisting citizen scientists at each sites for the first three flights. The principle issue is the location of the portable GCPs which can be affected by citizen scientists’ spatial effort bias^31^, leading to positional errors due to GPS signal blockage from foredunes, tall trees or buildings. As GCPs positional errors directly impact the point cloud georeferencing process (hence, the resultant DSM), we assessed whether significant (p = 0.05) differences exist in GCPs spatial dispersion (i.e. their two-dimensional spread across the surveyed area), their positional X, Y and Z variances and the images georeferencing accuracies (X, Y and Z rmse) reported by the photogrammetric software Pix4Dmapper (V4.3.31), between locations (Fig. 2).

The Kruskal–Wallis H test^49^ indicated that there are statistically significant differences (p = 0.05) in all positional GCPs variances (Fig. 2a) and in image georeferencing errors (Fig. 2b), between the different locations. This can be observed in the pairwise comparison heatmaps (Fig. 2c–e), where in the great majority of cases, statistically significant differences in X,Y and Z variances are found between any one location and at least four others. This is likely to be the cause of all locations having statistically significant differences in rms georeferencing errors compared to each other (Fig. 2f). Importantly, GCPs elevation precisions were outside acceptable variance (20 mm) 7% of the time (61 out 886), with only 9 cases (1%) exceeding 64 mm (outliers). Therefore, positional precision differences between locations are largely attributed to random errors during the GCPs position recording process and no citizen scientist’s bias can be ascertained. The same applies to the images georeferencing errors. Their location-specific medians range from a minimum of 2 mm (Seaspray and Cowes) to a maximum of 15 mm (Warrnambool), which are of the same order of magnitude of the GCP positional precisions. As such, we cannot exclude the possibility that these differences are also due to random errors in the geolocation recording of the GCPs.

Regarding the GCPs two-dimensional spatial spreading (Supplementary Fig. S6), the Kruskal–Wallis H test showed that there are no statistically significant differences between locations (H = 11.12; p = 0.28, n = 78).

Additionally, to assess whether there are statistically significant differences between the end-products of our protocol across locations (i.e., the expected DSM error) and considering that more independent checkpoint surveys were unavailable, we analysed limits of detection thresholds as indicators of ‘overall’ data quality (Supplementary Method “Limit of detection analysis”). An example of limit of detection threshold derivation and error normality evaluation in Apollo Bay is shown in Supplementary Fig. S8. The Kruskal–Wallis H test found that no significant differences (H = 16.167, p = 0.063, n = 73) exist between each location specific threshold distributions at the 0.05 significance level.

### State-level volumetric monitoring of open-ocean and embayed beaches

**Figure 3:**
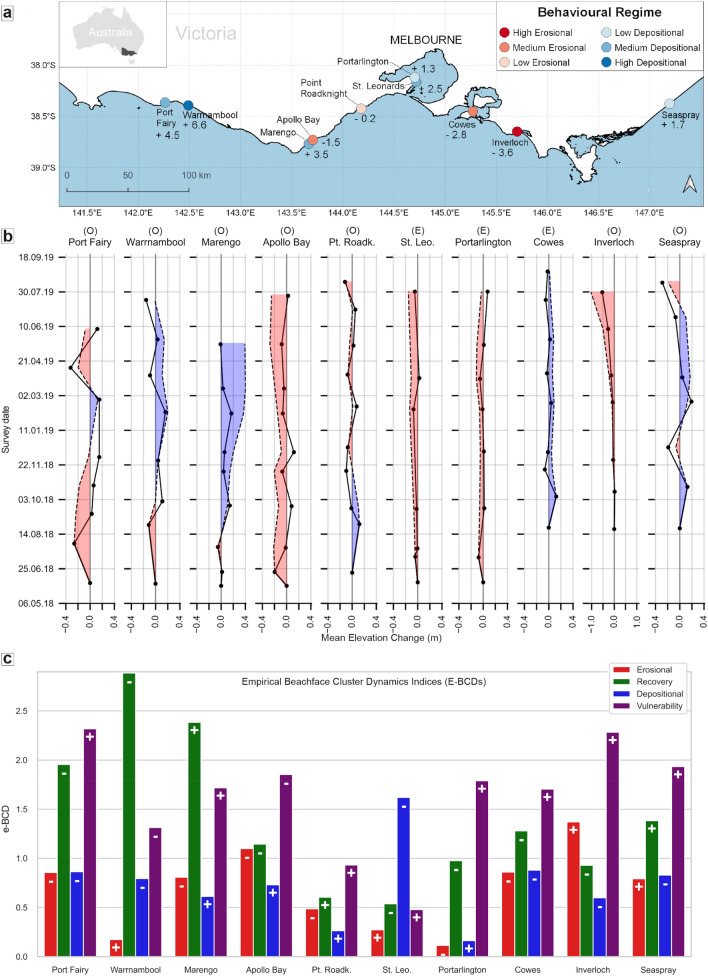
Citizen scientists allow for the quantification of beach volumetric and hotspot dynamics in Victoria, Australia. (**a**) Locations and behavioural regimes (residual beachface cluster dynamics index, r-BCDs) of citizen science monitoring sites. Classes obtained using Jenk’s natural breaks classification^50^. (**b**) Mean elevation change (MEC, in meters) across open-ocean (marked with “o”) and embayed (marked with “e”) sandy beaches. The solid line shows the inter-surveys MEC while the dashed line indicates its cumulative value since the beginning of monitoring. Red and blue areas highlight periods of negative and positive sediment budgets relative to the beginning of the monitoring, respectively. Beachface recovery time can be estimated as the time the cumulative MEC takes to reach zero after erosional events. Please note the different scale for Inverloch for display purposes. (**c**) Empirical beachface cluster dynamics (e-BCDs) indices showing site erosional, depositional, recovery and vulnerability behaviours during the monitoring period. Signs indicate increasing (+) and diminishing (−) magnitude trends.

Overall, 100,794 ± 243 m^3^ of sand has been transferred off the beachfaces (net erosion) during the monitored period (from the first June 2018 to the 29th August 2019) across the 10 locations (Fig. 3a, see Additional Method “Area of study” for more information). Swell-exposed open-ocean beaches displayed mean elevation changes (MEC) of greater amplitudes than embayed beaches, which are situated along fetch-limited or sheltered coastlines. All the following observations are relative to the whole aforementioned monitoring period.

On average, the absolute MEC for open-ocean beaches (0.11 ±  < 0.01 m) is almost three times larger than for embayed ones (0.04 ±  < 0.01 m). During erosional phases (negative MEC in Fig. 3b), the average MEC is − 0.13 ±  < 0.01 and − 0.04 ±  < 0.01 m in open-ocean and embayed beaches respectively. Similarly, during recovery phases (positive MEC in Fig. 3b), the average MEC is + 0.09 ±  < 0.01 and + 0.04 ±  < 0.01 m on open-ocean and embayed beaches respectively. Post-erosion recovery times are highly variable, ranging from a few days (Warrnambool) to approximately one year (Portarlington), while some locations never fully return to their initial state (Apollo Bay, Inverloch).

To model the monitored beachface dynamics and distinguish between erosional and depositional behavioural regimes, we used the residual beachface cluster dynamics index (r-BCD, Method, Fig. 3a). Therefore, a location r-BCD index is one single signed value that characterises the dominant behaviour that the beach in that location exhibited during the entire monitored period.

The open-ocean beaches of Port Fairy, Warrnambool, Marengo and Seaspray exhibit highly to slightly depositional regimes. Interestingly, Port Fairy and Seaspray r-BCDs indicated that both locations had a depositional behavioural regime during the monitoring period despite these locations experiencing net sediment losses of − 10,962 ± 66 m^3^ and − 7,517 ± 726 m^3^ respectively. In other words, their intra-annual cut and fill dynamics (Fig. 3b) are skewed toward net sediment gains. By contrast, Inverloch, Apollo Bay and Point Roadknight display highly, moderately and slightly erosional behavioural regimes which are corroborated by end-of-monitoring sediment losses of − 66,700 ± 909 m^3^, − 13,871 ± 2,286 m^3^ and − 2,159 ± 562 m^3^ respectively.

The embayed beaches of St. Leonards, Portarlington and Cowes are protected from the predominantly south-westerly swell being located within Port Phillip and Western Port Bays. Despite Portarlington never fully recovering from its first erosional event in the timeseries, its depositional behavioural regime indicates that it is more likely to accrete rather than erode. Conversely, Cowes displays a highly erosional behavioural regime despite never showing a negative sediment budget respective to the beginning of the monitoring.

### Sediment dynamics highlight accelerated or slowed-by-intervention erosion in critical locations

Empirical beachface cluster dynamics indices (e-BCDs, Fig. 3c) measure the importance (absolute score) and main magnitude trend (score sign) of beachface erosional, recovery, depositional and vulnerability behaviours. We use e-BCDs to detect naturally accelerating and slowed-by-intervention erosional modalities in Inverloch and Apollo Bay respectively. The e-BCD indices are representative of the whole monitoring period and are intended to depict the main trend of beachface sediment dynamics that occurred during the observation time.

From the 22th August 2018 to the 30th July 2019, the monitored area in Inverloch displayed an erosional score (+ 1.37) that is greater than its high recovery score (− 0.93), indicating that erosional hotspots mostly continued to erode the beachface rather than recover. The combination of positive erosional and negative recovery indices imply that through time, an increased amount of sediment has been eroded, while recovering areas tended to accumulate less sediment than was previously lost. The depositional score (− 0.60) is substantially lower than the vulnerability (+ 2.28), indicating that depositional areas became erosional, rather than continuing to accrete. These signs suggest that depositional hotspots gained less sediment through time and, once starting to erode, the volumes lost were typically higher than what had previously been deposited.

These dynamics indicate an accelerated beachface depletion, which is confirmed by Inverloch mean elevation change time series (Fig. 3b). An accelerating erosional phase started in mid-October 2018 and lasted until the end of the monitoring, totalling a net loss of 48 ± 0.06 m^3^/m, at a rate of 0.14 m^3^/m eroded daily, beach wide.

**Figure 4:**
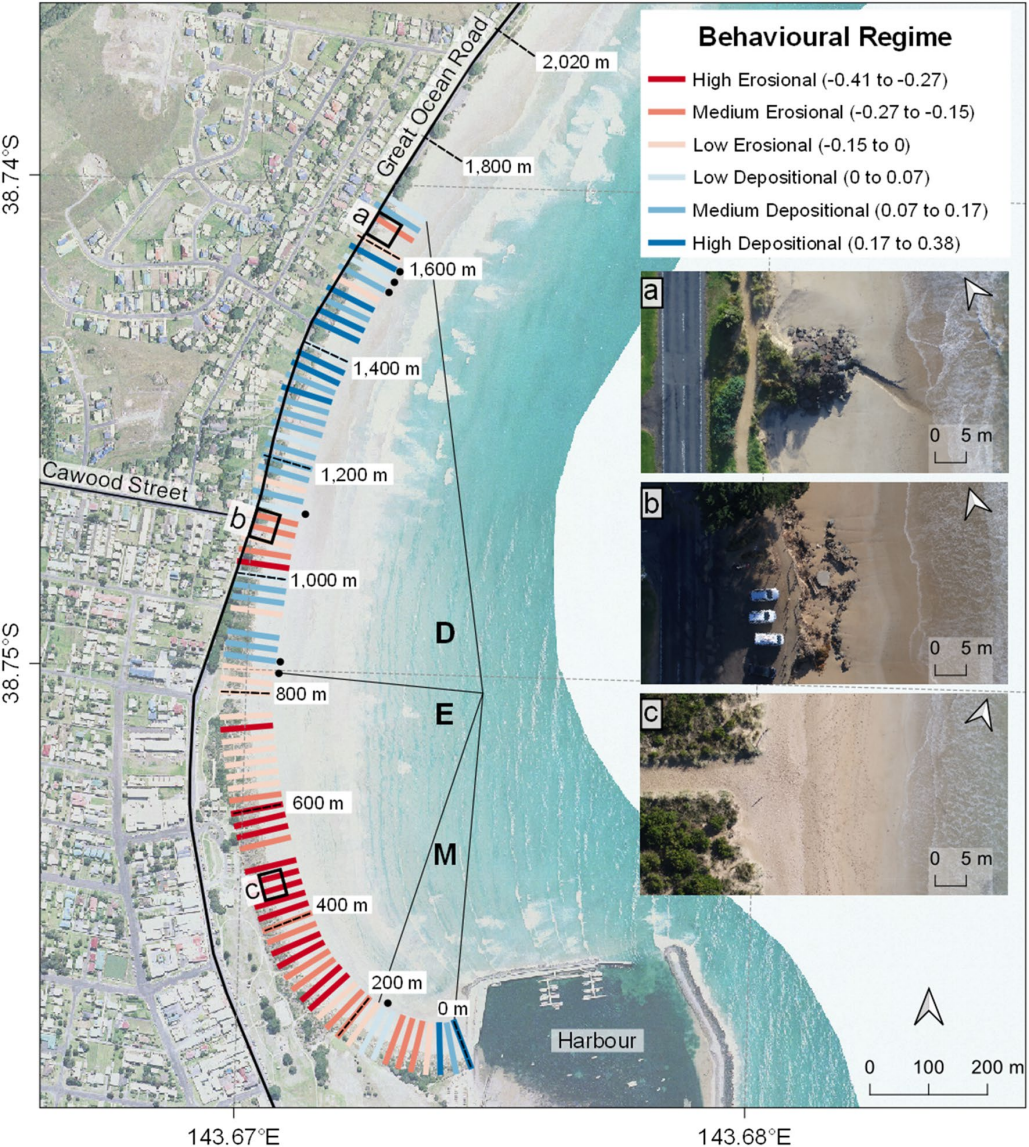
Behavioural regimes at transect level showing the success of sand nourishment. Apollo Bay transect-specific behavioural regimes (r-BCDs) highlights a marked transition from mostly erosional behavioural regimes (180 to 840 m alongshore from the harbour, zone E) to mostly depositional ones (beyond 840 m alongshore, zone D), which corresponds to the areas where most of the nourishment sand has been deposited and reworked by the northward longshore drift. Dashed transects mark the distance alongshore displayed next to them. Please note this map displays behavioural regime classes as derived from transect-specific r-BCD index (displayed in parenthesis) using Jenk’s natural breaks classification^50^ and do not represents elevation changes. The insets a, b and c represent a medium erosional area due to a storm water drain in zone D, a medium erosional area despite nourishment in zone D and the main beach entrance and highly erosive area in zone E respectively. Only transects that consistently had a minimum of 10 valid points for at least 7 of the 9 time periods available in Apollo Bay have been retained. The minimum period threshold (7) and minimum valid points (10) per transect have been chosen after a sensitivity analysis (Supplementary Discussion “Apollo Bay behavioural regime sensitivity analysis”). Dots signal transects that changed sign (from erosional to depositional or vice versa) as a consequence of the step minimum threshold and are considered less reliable. Map created with QGIS 3.4.0 (Madeira) using WGS84 coordinate system.

In Apollo Bay from the first June 2018 to the 25th July 2019, the beachface dynamics have been impacted by a weekly sand nourishment program that deposited 16,050 m^3^ of sand in the intertidal and foredune areas (focussing from 800 m north, zone D in Fig. 4), from the 19th June to the 4th September 2018 and from the 22nd May to the 21st June 2019.

The e-BCDs analysis in Apollo Bay indicates that erosional hotspots tend to turn depositional by a slight margin (− 1.10 erosional and − 1.14 recovery scores), in which case a lower amount of sediment is deposited compared to what had been previously lost (negative depositional sign). On the other hand, depositional hotspots tend to turn erosional by a greater margin (− 1.85 vulnerability and + 0.73 depositional scores), in which case the sediment lost is usually less than what was previously deposited. Overall, Apollo Bay e-BCD indices indicate a slight erosional predisposition (driven by the vulnerability index), precariously reduced by “sediment-sparing” mechanisms (negative signs of erosional and vulnerability e-BCDs) that slows the beachface sediment loss. In fact, despite being supported by sand nourishment, Apollo Bay sediment volumes never returned to its initial state at the commencement of the surveys. This indicates that the management intervention in Apollo Bay is likely to be slowing beachface erosion during the monitoring period, hence, we observed a slowed-by-intervention erosional mode.

### Site level case study: assessing beach nourishment efficiency in Apollo Bay

To demonstrate the scalability of citizen scientists’ data, we down-scaled behavioural regime, morphological and volumetric analysis from the location to the single transect level, assessing the efficiency of a beach nourishment project in protecting backshore economical assets.

The Great Ocean Road is a scenic coastal drive that contributes up to 6.1% of revenue to the regional economy^51^. It attracts more than 5,000,000 visitors annually, and Apollo Bay is the second most popular visitor destination^51^. The spatial distribution of transect-specific r-BCDs indicates three distinctive zones where behavioural regimes are mostly mixed (M), erosional (E) or depositional (D) (Fig. 4).

**Figure 5:**
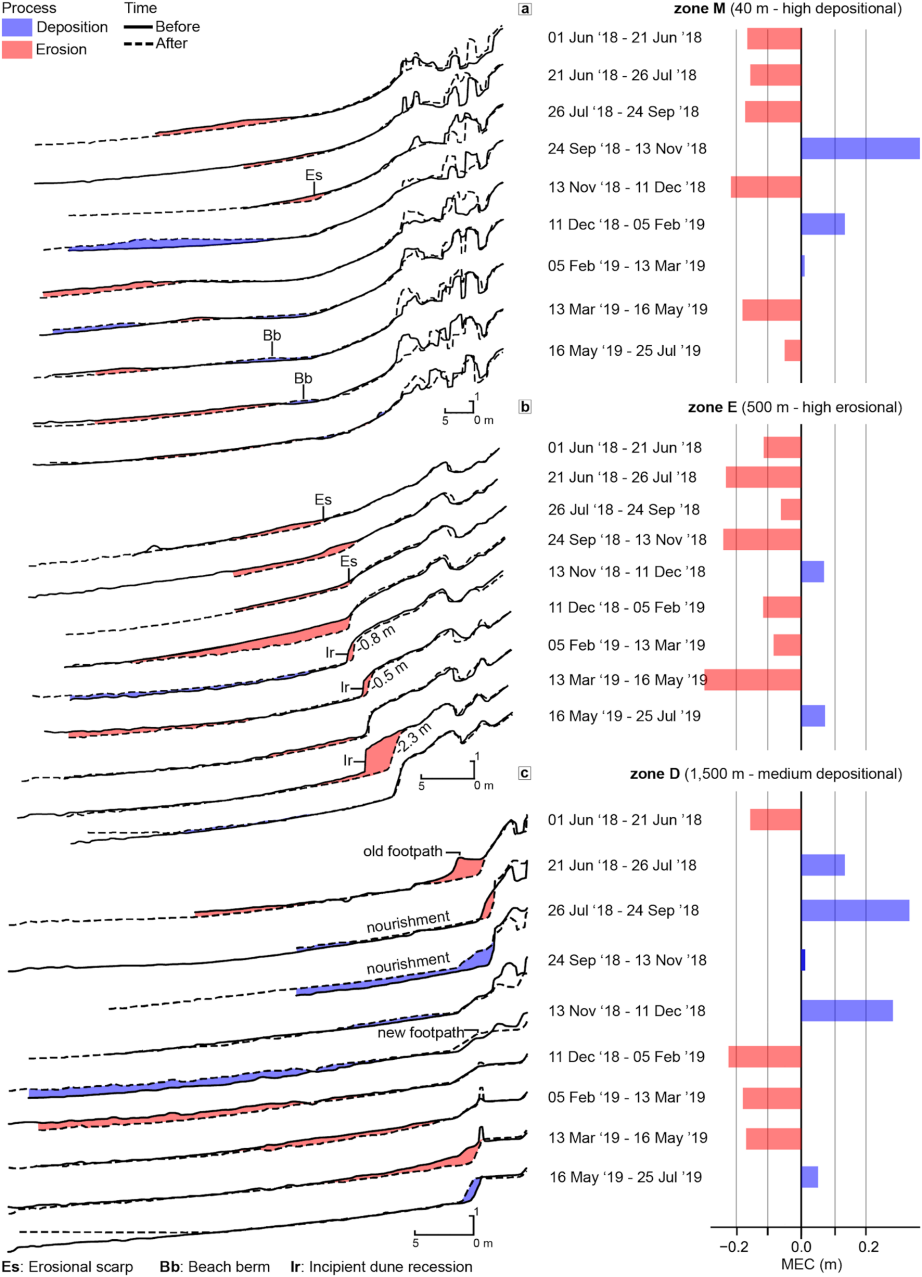
Representative cross-shore profiles in zones M, E and D exemplify distinct morphological dynamics. (**a**–**c**) Show before (solid line) and after (dashed line) elevation profiles in zones M, E, D, respectively. The time period considered is displayed between the profiles and the mean elevation change (MEC) histogram plots, which show the transect net beachface lowering (red) or raising (blue) of each period, considering limits of detection. The distinction from zone M and E could be attributed to timing and magnitude of depositional events, as the erosional ones show relatively similar mean elevation change.

Zone M represents a mixed-zone extending from immediately in the lee of the harbour to 180 m alongshore. Generally, erosion during the monitoring period occurred on the lower intertidal beach and occasionally on the incipient dune, leaving the vegetated foredune intact. Swash-generated berms (Fig. 5a) occur throughout zone M and are typical landforms of low tide terrace type beaches^52^.

Zone E is predominantly erosional, despite this area being reported to have experienced accretion since 1956 due to the construction of the harbour and its shadowing effect from incident waves^53^. Here, the erosion in the intertidal zone is mostly uninterrupted throughout the surveys, leading to a gradual transgression of the erosional scarps, eventually causing up to 6 m of incipient dune recession (Fig. 5b). The most erosional transects are located 500 m alongshore, within 40 m from the main pedestrian beach access (Fig. 4 inset map c). Further to the north, the magnitude of beachface lowering gradually diminishes to finally become net accretion accompanied with foredune recession at 800–840 m alongshore, entering zone D.

Zone D has been reported to be receding since mid-1980^53^ and is also where the majority of nourishment has occurred during the monitoring. Notwithstanding, zone D comprises two “erosional enclaves” (Fig. 4 inset maps a,b) which signal local erosional hotspots of major concern. Moreover, despite an informal rockwall effectively protects the foredune between 1,100 and 1,200 m alongshore, foredune recession has been widespread between June and July 2018 causing sections of a footpath to collapse (1,500 m alongshore, Fig. 5c).

**Figure 6:**
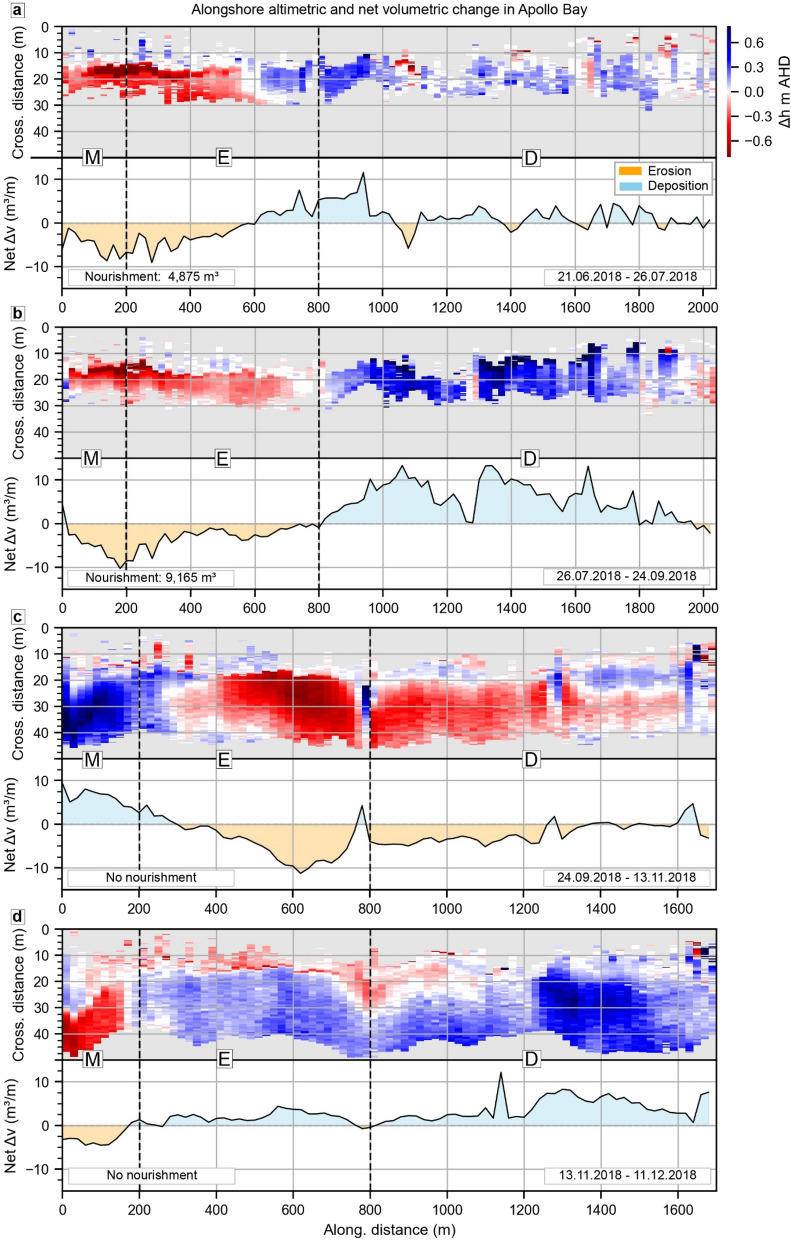
Selected periods in Apollo Bay when volumetric change best express behavioural regime zones M, E and D. Apollo Bay inter-surveys alongshore variation of altimetric change (top parts, in meters from Australian height datum, AHD) and net volumetric change (bottom parts, in cubic meters per meter of beach length, m^3^/m). Only sampling points in the beachface which have been classified as sand and are not within limit of detections are retained. Grey areas represent either swash, no-sand or areas beyond the landward baseline (0 m cross-shore). White areas are changes within limit of detections thresholds. Cross-shore distances refer to the distance of each observation from the landward baseline, which is defined by either 2–3 m beyond the vegetation line or by human infrastructures (e.g. footpaths). Alongshore distances refer to the location of each transect from Apollo Bay Harbour, while the bold M, E and D letters partition the behavioural regime zoning.

The different behaviours in zones M, E and D can be observed by adopting a beach-wide perspective, while considering the volumes of sand introduced by the nourishment program. From the first of June 2018 to the 21th June 2018 (Supplementary Data ‘site_level_change”), particularly stormy weather eroded 9,055 ± 76 m^3^ of sand from the beachface, slightly alleviated by post-storm sand renourishment of 1,320 m^3^. From the 21th June to 26th July 2018 (Fig. 6a) the beachface recovery was assisted by 4,875 m^3^ of renourished sand, taking the alongshore volumetric change in this period only slightly below zero (− 0.20 ± 0.09 m^3^/m). The majority of deposition took place in zone D where renourishment focussed, while zone E kept losing sediment, especially from the intertidal beach (Fig. 5b). This pattern is accentuated in the next period (from 26th July 2018 to 24th September 2018, Fig. 6b) when 9,165 m^3^ of sand was supplied to the beach by managers mostly northward from the 800 m mark (Fig. 4).

During these renourishment periods, zones E and D behaviours are noticeably inverse to each other, whereas zone M generally follows the behaviour of the adjacent zone E. When renourishment is suspended, zones E and D are essentially undifferentiated, whereas zone M is remarkably dissimilar to the rest of the beach, especially during the spring to summer transition (from the 24th September 2018 to 11th December 2018, Fig. 6c,d).

Overall, after initial recession of the foredune, the intertidal zone showed a net accretion by the end of the monitoring, indicating that the depositional behaviour of zone D is precariously tied to the nourishment operations. In fact, during nourishment periods, the alongshore distribution of volumetric change clearly sets zone D apart, being it the only accreting zone amongst overall beach erosion (Fig. 6a,b). Conversely, when nourishment is suspended, zones E and D erode or accrete conjointly, while zone M shows a noticeably divergent behaviour (Fig. 6c,d).

Therefore, in the absence of nourishment, we would expect the whole monitored area in Apollo Bay to be erosional, corroborating our previous argument about the slowed-by-intervention erosional mode of Apollo Bay. We can conclude that the current sand nourishment strategy is likely to be locally decelerating a recent beach wide erosive trend, but it is not sufficient to maintain or fully recover subaerial beach sediment budget in the long-term. Additionally, mostly depositional areas seem to be accreting the intertidal beach, while the foredune is still receding.

## Discussion

The vertical accuracy of the citizen scientists’ protocol (rmse = 0.089 m) obtained in an operational scenario is 0.03 m less accurate than the median accuracy reported across the relevant UAV-SfM beach monitoring literature (median rmse = 0.059 m). However, using robust statistical estimators which put less weights on error outliers (focussed around foredune vegetation) led to an error estimation of 0.048 m, slightly better than what others obtained in similar works^27,54^ (Supplementary Discussion “Citizen scientists’ accuracy”). Our bias analysis indicates that citizen scientists’ ground control points (GCPs) spatial arrangement was consistent through time and space. GCPs positional precision and image georeferencing errors have significant inter-groups differences which cannot be attributed to citizen scientists as errors are below the reported GCPs precision. As no significant differences of limit of detection distributions between locations have been found, we can assert that limited bias impacted the quality of data during the full data creation pipeline, with our protocol. Therefore, as citizen scientists' data is of comparable accuracy and bias to professionally acquired UAV-SfM datasets, it is considered of high-quality^36^.

Besides monitoring beachfaces volumetric changes, our approach temporal resolution allowed us to conceive the residual beachface cluster dynamics index (r-BCD) to model hotspots behavioural regimes in the absence of precise wave data, but based on topography alone. The fundamental assumption of r-BCDs is tied to the classic morphodynamic feedback loops of process and morphology^55^. As r-BCDs are derived from high frequency elevation changes, they incorporate the morphological variability that landforms experienced due to the locally active geomorphic processes. Citizen scientists allow r-BCDs to be used over multiple locations at site and transect scales, providing government authorities a rapid and powerful beach dynamics assessment that managers can use to prioritise erosion hazard mitigation expenditure. This approach becomes especially favourable in situations where high quality nearshore waves and currents data is lacking and can complement other cost-effective remote sensing approaches, such as satellite-based coastal monitoring.

Multispectral imagery acquired by optical satellites, such as Sentinel-2 or Landsat, allows the use of two-dimensional shoreline landward/seaward shifts as erosion/deposition proxy for large scale erosion monitoring. However, shorelines derived from space are difficult to validate in-situ, consequently, only a few studies used ancillary beach topographic data or coincident shoreline GPS surveys to test the accuracy of the extracted shorelines^56–59^. Moreover, sandy beaches topographic features such as beachface slope^60^, intertidal extent^61^ and elevation^62,63^ at continental-scale have also started to be derived from space-based observations. These methods need beach topographic data at higher resolution to be validated against. Our study demonstrates that citizen science UAV-SfM is in an advantageous position to provide the high-quality and reliable 3D data that is essential for ground truthing space-based observations.

Alternatively, the closest comparable topographic monitoring methods are repetitive airborne light detection and ranging (LiDAR) surveys or fixed coastal imaging stations (ARGUS). Coastal LiDAR is often used for quantifying wide-scale shoreline spatial variability of single-event post-storm erosion^19,64,65^, however, high operational costs have traditionally limited its temporal resolution within geoscientific research^66^, often missing the fine seasonal dynamics that our approach provides. ARGUS stations on the other hand can typically provide hourly shoreline images of key sites, which are then processed to obtain shoreline elevations and later used to monitor erosion/accretion patterns in a relatively cost effective way. However, ARGUS-derived shoreline elevations are computed with a variety of empirical, semi-empirical and complete numerical models, depending on the quality and amount of site-specific field data (topography, tide and offshore wave measurements) used to calibrate the shorelines. As a consequence, vertical accuracies (in the range of 0.1 to 0.4 m) and methodologies are often site-specific^17^, possibly limiting the integration of multi-site observations into one integrative study. With our approach, 10 UAVs and 100 smart ground control points were used to repeatedly survey (7 to 10 times) 10 key locations at a total cost of US$250,590 (including equipment, online data processing and hosting and the time to train and support a group of 3–4 citizen scientists per location). This equals to US$3,020 per survey (n = 83), which is expected to be approximately US$1,290 per survey in the next years, as groups become established and less training and support hours are budgeted (Supplementary Discussion “Monetary costs''). Therefore, we consider our approach to be cost-effective as high-quality, high temporal and very high spatial resolution beach topographic monitoring is consistently achieved at 10 high-priority locations (site scale), which are representative of the Victorian coast (regional scale).

The cost-effectiveness and geographical coverage offered by our approach has important implications in contexts of erosion mitigation in vulnerable communities, such as those in small island developing states (SIDS). In SIDS, a lack of quality coastal data at relevant spatiotemporal scale has been recognised^67,68^ and their economic dependence to tourism and coastal eco-services within a changing climate context put their subsistence at risk^69^. Our approach would allow SIDS communities to reliably monitor coastal erosion in sensitive areas cost-effectively, producing continuous survey-grade topographic data over wide and often dispersed geographic areas, with some limitations.

Without environmental context information (especially wave climate), our methods reliably depict seasonal beach change trends but cannot explain the geomorphic forcings responsible for those changes. In fact, the magnitude of changes could differ depending on how far in time from major storms (not investigated here) the surveys are. Although we demonstrated the reliability and usefulness of our approach, coastal processes data is needed to formulate more causative sand dynamics and geomorphological interpretations, which would allow more accurate sand dynamics to be evaluated. This is the next logical step and focus of future studies.

Additionally, as opposed to other coastal citizen science projects where anyone can take part as a citizen scientist^70,71^, in our approach, legislation plays a central role in defining who can be a pilot, directly challenging its feasibility and reproducibility around the world (Supplementary Discussion “Role of legislation”). In general, the key legislation requirements that need to be met in order to perform and replicate our protocol are that (1) UAV flight are permitted over interest areas, (2) automatic (waypoint-based) UAV flight mode is allowed and (3) no mandatory UAV flight license is required for research applications using sub-2 kg airframes.

In Australia, scientific operations with UAVs below 2 kg of weight are part of the “sub-2 kg excluded category”, which allows individuals older than 16 years of age to fly small UAVs (1) within standard operating conditions, (2) without the need of a remote pilot licence and (3) without a mandatory risk assessment to be approved. Elsewhere, various UAV laws can challenge, impede or allow UAV citizen science applications. In SIDS, UAV regulations range from total ban (Cuba and Barbados) to “Australia-like” approach (Papua New Guinea), with important variants that limit the feasibility (heavy air-traffic and multiple authorities permit systems in Maldives) or replicability (automatic flight procedures not allowed in the Dominican Republic) of our protocol. On the other hand, from the 31st December 2020 the European Union Aviation Safety Agency (EASA) UAVs regulation will allow UAV citizen science projects to take place in 32 European countries (25 of which are coastal), thanks to a regulation very similar to the one adopted in Australia. Despite the combination of a greatest number of coastal no-fly zones (due to controlled aerospace from the military, proximity to aerodromes or natural protected areas) with crowded beaches can discourage UAV citizen science coastal applications in EASA countries, our protocol can also be applied to other environments or scientific disciplines where accurate cost-effective topographic monitoring is needed. In fact, multi-temporal UAV-SfM has already been used by professional researchers around the world for monitoring erosion in mudflats^72^, badlands^73^, agricultural watersheds^74^, rivers^75^ and open-pit mines^76^. Additionally, UAV-SfM topographic data has also been used for non-erosion purposes to monitor both natural processes, such as landslide dynamics^77^, sediment retention dams filling^78^, crops growth variability^79^, forest trees growth^80^, snow depth^81^ and glaciers melting dynamics^82^, or anthropogenic processes, such as landfills growth rates^83^, environmental contamination^84^ or hiking trails conditions^85^.

In conclusion, our results not only demonstrated the value of citizen scientist’s high-quality and unbiased data for multi-scale sandy beaches sediment dynamics monitoring, but can also encourage further application of citizen science with drones into all the aforementioned scientific applications. This not only would greatly expand the spatiotemporal scale of scientific experiments, but also democratise scientific engagement access, enhance global environmental awareness and transform citizen scientists into key stakeholders within an adaptive environmental management system.

## Methods

In this work, we used machine learning and limit of detection analysis to detect and reliably quantify subaerial beachface dynamics due to sand-only and above detection limits changes. Spatial autocorrelation analysis is also employed at the site-level to remove spatial outliers and detect statistically significant (p = 0.05) hotspot of changes to better characterise sediment dynamics through time. This information is then explored with novel indices (empirical and residual beachface cluster dynamics indices, e-BCD and r-BCD respectively) based on discrete Markov chain models, at the site and transect scales.

### Virtual network of elevation profiles

A virtual network of digital transects was created for every site and kept fixed during the analysis. Transects are uniformly distributed alongshore with a spacing of 20 m, normal to the shoreline, with an across-shore length of 80–150 m, depending on the beach width discernible from the earliest orthophoto available (Supplementary Fig. S1). We extracted synchronous elevation and colour information along each transect, with a 0.1 m sampling step, in all 10 sites and surveying dates (8 to 10 surveys per site), resulting in a total of 6,809,610 points on 999 transects.

Swash zones were excluded from the analysis. We only retained points which were classified as sand and within the subaerial beachface, which is defined as the area from the upper swash to 2–3 m landward of the vegetation line or where anthropogenic barriers are present.

### Machine learning sand classification

We used machine learning to restrict the analyses to those extracted points that are sand, removing the ones representing beach wrack or coastal vegetation that would otherwise skew our volumetric and behavioural computations. For each survey, we performed the Silhouette Analysis^86^ to find a sub-optimal number of clusters (*k*) to partition the points with KMeans clustering algorithm^87^, using spectral (red, green, and blue bands) and topographic (slope, curvature and distance from the transect seaward origin) features. By iteratively run KMeans (parameters: initial cluster centres selected with KMeans ++, 300 iterations per run, inertial tolerance of 0.001, pre-computed distances) increasing *k* by 1 at every iteration (up to *k* = 20), we were able to compute the overall silhouette coefficient associated at every *k*. We chose as sub-optimal *k* the value after which a greater *k* would not substantially reduce the overall clustering performance. Once the sub-optimal *k* has been found for every dataset and KMeans run using it, clusters were displayed in QGIS (version 3.2.3) and visually labelled as sand or no-sand (Supplementary Fig. S1). No-sand points across each transects have been replaced by an interpolated value using a linear model. Minor manual editing was required to correct sand points erroneously assigned to a non-sand cluster. This mainly occurred in very dark shadows cast by tall trees along coastal walking paths or right below near vertical foredune.

### Limit of detection thresholds

The morphological method^88^ has been used in a variety of environments, including sandy beaches^23,29,31,89^. It involves the subtraction of two digital surface models (DSMs) of the same location at different times to obtain a DSM of Difference (DoD), which represents surface change over a shared elevation datum for each period. Our transects are subject to the same error estimation techniques used for DoD analysis. Therefore, in order to account for areas of apparent elevation change (Δ*h*) due to inherent DSM uncertainties, we computed limit of detection thresholds for each DoD (n = 78). Changes within these thresholds are considered uncertain and their contribution is expressed as error margins when altimetric or volumetric change is reported.

To obtain limit of detections, we firstly identified invariant objects as close as possible to the beachface in each site (paths, roofs, rockwall, anthropogenic structures), which we used as calibration zones. Then, we manually digitised vector lines across calibration zones and created a checkpoint every 0.1 m along those lines, resulting in 23,660 check points. Finally, we measured the checkpoints Δ*h* in each DoD and obtained the threshold computing the normalised median absolute deviation^45^ as robust statistical estimator.

### Volumetric computations and mean elevation change

The exclusion of points within the swash extent and variations in UAV survey coverage resulted in irregularities in the number of transects and valid points per survey (see Additional Data). Therefore, we compared subaerial changes across sites and time using the mean elevation change (MEC), as follows:

$$Mean\,elevation\,change \left( {MEC} \right) = \frac{1}{n}\mathop \sum \limits_{z = 0}^{n} (z_{post} - z_{pre} ),$$

where *n* is the total number of valid elevation points, $z_{pre}$ and $z_{post}$ are the elevation above Australian height datum (equivalent to mean sea level) values occurring at the same location in both pre and post surveys.

Additionally, when no inter-site comparisons were involved, we approximated the alongshore volumetric change (in m^3^/m) as:

$$Along. beachface \,change = \mathop \smallint \limits_{{x_{swash} }}^{{x_{limit} }} \left( {z_{post} - z_{pre} } \right)dx,$$

where $x_{swash}$ and $x_{limit}$ are the upper swash and landward limit respectively. Plus or minus ( ±) error intervals for both MEC and volumetric change estimates represent the uncertainty related to changes within the limit of detection thresholds.

### Hotspot analysis: local Moran’s I

In order to obtain spatially explicit and statistically significant hotspots of erosion or deposition at the site level, the Local Moran-I^90^ ($I_{i}$) statistics with false discovery rate correction was computed for every inter-surveys elevation difference (Δ*h*) points. The $I_{i}$ statistics is defined as:

$$Local Moran^{\prime}s Index \left( { I_{i} } \right) = \frac{{z_{i} - \mu_{z} }}{{\sigma^{2} }}\mathop \sum \limits_{j = 1, j \ne i }^{n} \left[ {w_{ij} \left( {z_{j} - \mu_{z} } \right)} \right],$$

where $z_{i}$ is the value of the variable at location *i* with $\mu_{z}$ and $\sigma^{2}$ the respective mean and variance, as calculated on the $n$ number of observations; $w_{ij}$ is the spatial weight between the observation at location *i and j* and $z_{j}$ is the value of the variable at all locations different than *i*^90,91^.

A binary row-standardised spatial weight matrix conceptualises spatial regions (neighbourhoods) within a 35 m of radial Euclidian distance from each focal point, obtaining the weights $w_{ij}$ for all points in relation to each other. The 35 m distance band has been chosen to include in the neighbourhood sand points from the two adjacent transects. We used 999 random permutations to compute the reference distribution and obtain a minimum pseudo p-value of 0.05 (95% level of confidence), which we used to discard non-significant $I_{i}$.

In this analysis, only significant High-High (areas where high values are surrounded by high values) and Low-Low (areas where low values are surrounded by low values) hotspots have been retained, discarding spatial outliers.

Significant hotspots of Δ*h* have been classified into five magnitude classes, using the Jenks-Caspall optimised-natural breaks method^92^, based on the totality of absolute Δ*h* values (Table 1). Weights of each magnitude class are used to represent the severity of change during the e-BCD sign computations and are obtained deriving the median absolute values for each Δ*h* magnitude class.

**Table 1:** Elevation change classes used as transient states for BCD indices computation.

Magnitude	Weight	Erosional		Depositional	
		Limits (m AHD)	Label	Limits (m AHD)	Label
Undefined	0.10	0 to − 0.15	ue	0 to 0.15	ud
Small	0.20	− 0.15 to − 0.27	se	0.15 to 0.27	sd
Moderate	0.34	− 0.27 to − 0.44	me	0.27 to 0.44	and
High	0.54	− 0.44 to − 0.72	he	0.44 to 0.72	hd
Extreme	0.90	Below − 0.72	ee	above 0.72	ed

Undefined classes are so named due to being close to global LoD (0.05 m AHD), even if elevation changes within  ±0.05 m AHD have been removed.

*AHD* Australian height datum, approximatly mean sea level.

### Empirical and residual beachface cluster dynamic indices

The empirical and residual beachface cluster dynamics indices (e-BCD and r-BCD respectively) are purposefully designed metrics to leverage the very high spatiotemporal resolutions and three-dimensionality of our data for studying subaerial beach landform dynamics (morphodynamics). With elevation change (Δ*h*) magnitude classes as transient states (Table 1), we used finite discrete Markov chain models to compute first-order stochastic transition matrices and steady-state probability vectors, used to derive e- and r-BCD respectively.

Following Lambin (1994), a discrete Markov process can be represented as:

$$s_{t + 1} = Ms_{t} ,$$

where $s_{t}$ is a column vector, $s = \left( {s_{1} , \ldots ,s_{m} } \right)$ having as elements the valid points (within the beachface and beyond LoD sand-only Δ*h* points) in one of the $m$ states (i.e. Δ*h* magnitude classes) at time $t$. M is a $m \times m$ matrix holding the first-order (from $t$ to $t + 1$) transition probabilities $p_{ij}$, derived as:

$$p_{ij} = \frac{{n_{ij} }}{{\mathop \sum \nolimits_{k = 1}^{m} n_{ik} }},$$

where $n_{ij}$ is the number of transitions from an initial state $i$ to state $j$ and $m$ is the number of states (i.e. elevation change classes in Table 1) in which each observation can be. The matrix M is row-standardised, so that the sum of transition probabilities from a given state is always equal to one.

The e-BCDs divide the first-order transition matrix M into four sub-matrices (Supplementary Fig. S7), each representing site-level erosional, depositional, recovery and vulnerability behaviours of the subaerial beach over the monitoring period. The e-BCDs indices are computed for every sub-matrices ($sub$) as follows:

$$Empirical\, BCD_{sub} = \mathop \sum \limits_{i,j = 1}^{n} \left[ { ws^{\prime}_{ij} } \right]\times p_{ij} ,$$

where $ws_{ij} ^{\prime}$ is a transformed weight that reflects the magnitude trend of such transition, which is defined as:

$$ws^{\prime}_{ij} = \left\{ {\begin{array}{*{20}l} { ws_{i} \times\left( { - ws_{j} } \right) \leftrightarrow i > j } \\ {ws_{i} \times ws_{j} \leftrightarrow i \le j } \\ \end{array} } \right\},$$

where $ws_{i}$ and $ws_{j}$ are the weights (Table 1) related to the initial $i$ and transitioning $j$ states respectively. The brackets “[ ]” indicate that the $ws_{ij} ^{\prime}$ transformation is implemented separately to determine the e-BCD sign only. The e-BCD absolute score computation does not implement this multiplication, capturing the process importance only. Any state to either no-hotspot or no-data transition probabilities are not included in the e-BCD interpretation.

The r-BCDs are computed from the steady-state probability vector.

The steady-state of a Markov chain returns a unique probability vector representing the states limiting probability distribution, which, once attained, one additional (or more) time steps will return the exact same initial states probabilities, signalling a situation of dynamic equilibrium has been achieved. This is represented as:

$$\pi M = \pi ,$$

where $\pi$  is the vector containing the limiting probabilities $\pi_{j}$ for each $j$ state in $s$. This vector $\pi$ is derived by solving a system of $m$ equations with $m$ unknowns, each equation represented as:

$$\pi_{j} = \mathop \sum \limits_{k = 1}^{m} \pi_{k} p_{kj} ,$$

given that:

$$\mathop \sum \limits_{j = 1}^{m} \pi_{j} = 1.$$

The steady-state can be seen in a descriptive way as representing the states hierarchy, which is unique to the system being modelled^93^, from which we derive the stochastic tendency the system had towards depositional or erosional states at the end of monitoring. We interpret this tendency as the most likely behavioural regime the system was subjected to, given the drivers of change and boundary conditions that influenced its evolution during the monitoring period.

The computation of the r-BCDs is as follows:

$$Residual \,BCD_{SS} = 100 \times \mathop \sum \limits_{i = 1}^{m} (\pi_{ie} - \pi_{id} ),$$

where $ss^{ }$ is the steady-state probability distribution of one location, $\pi_{ie}$ are the limiting probabilities of the erosional classes (ue, se, me, he, ee) and $\pi_{id}$ the limiting probabilities of the depositional classes (ud, sd, md, hd and ed) (Table 1). The r-BCDs are not signed as no transitions are represented in the resultant vector. Any state to either no-hotspot or no-data transition probabilities are not included in the r-BCD interpretation. The multiplication by 100 is performed for index readability purposes.

We computed r-BCDs using erosional/depositional hotspots at the site level only (Fig. 3a), while at the transect level (Fig. 4), r-BCDs are computed with the full Δ*h* cross-shore profiles. This has been done due to the relatively narrow beach width which resulted in a limited number of valid sand-only observations for hotspot analysis.

## Supplementary Information

Supplementary Information 1.

Supplementary Information 2.

## Data Availability

More than 220 3D datasets are already freely accessible to anyone via a user friendly web-platform to share and communicate information, promote coastal awareness, build knowledge and further increase the impact of our efforts. **Link** https://www.propelleraero.com/. **Credentials** email: vcmp@deakin.edu.au; Password: propellervcmp.

## Code availability

The code used throughout this study is freely available here: Draft code: https://github.com/npucino/sandpiper/tree/master. Except for digital photogrammetric procedure, all the analysis has been automated using open-source Python 3 packages, especially PySAL 2.0.0^93^ for LISA and Markov Chain computations, Scikit-Learn^94^ for machine learning classification and Seaborn and Matplotlib^95^ for quantitative data visualisation. Maps and some minor geoprocessing tasks have been accomplished using QGIS versions 3.0.2 (Madeira) and 3.2.3 (Bonn).
