# Supplementary material for: Citizen science for monitoring seasonal-scale beach erosion and behaviour with aerial drones
Source: Sci Rep. 2021 Feb 16;11:3935. doi: 10.1038/s41598-021-83477-6 (PMC7887256; doi:10.1038/s41598-021-83477-6)
Supplement: Supplementary file 1 — Supplementary Information 1. [file 41598_2021_83477_MOESM1_ESM.pdf]

Citizen science for monitoring seasonal-scale beach erosion and behaviour with aerial drones

Nicolas Pucino[1*], David M. Kennedy[2], Rafael C. Carvalho[1], Blake Allan[1], Daniel Ierodiaconou[1]

[1] Deakin University, School of Life and Environmental Sciences, Australia

[2] The University of Melbourne, School of Geography, Australia

*Corresponding author: npucino@deakin.edu.com

Supplementary Material

Supplementary Method "Area of study"

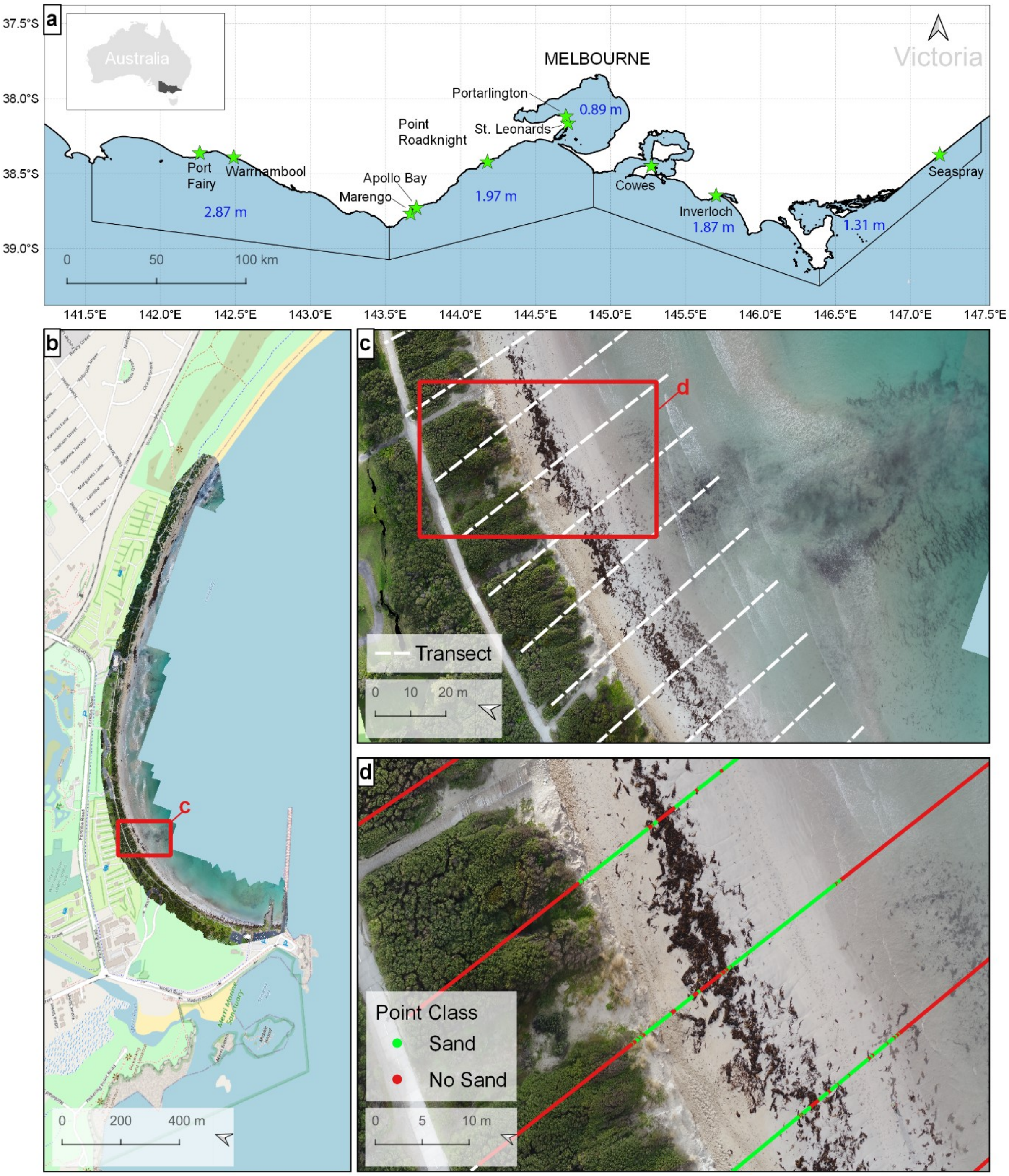

Suppl. Fig. 1. (a) Location of citizen scientists' surveying sites in Victoria. The coastal sectors represent the areas where the median significant wave height (blue values) have been calculated based on measurements from 01.01.1985 to 19.06.2020 of multiple radar altimeter satellites; (b) Survey extent example in Warrnambool. The UAV-SfM survey focuses on an area close to where backshore infrastructure are concentrated. The thematic basemap has been sourced from OpenStreetMap contributors at https://www.openstreetmap.org/copyright; (c) Cross-shore transects and beach wrack deposits; (d) Sand classification after machine learning procedure. UAV data collected in Warrnambool the 4th June 2018 following our protocol and accessible at https://www.propelleraero.com/ (email: vcmp@deakin.edu.au; password: propellervcmp).

In Victoria, Australia, ten sandy beaches (Supplementary Figure 1a) had been previously identified as being erosional hotspots using a combination of state-level multi-criteria analysis[1] and stakeholder consultation. Seven of these locations are open-ocean sandy beaches facing the predominantly south-westerly Southern Ocean swell, while the remaining three are situated along low-energy or fetch-limited coastlines, protected by headlands or estuaries. The orientations span from North to South through East with no beach facing West. Beach lengths vary from 5,800 (Port Fairy) to 700 m (Pt. Roadknight). The surveys do not cover the full beach length, rather, they focus on areas where backshore services and infrastructures are concentrated (Supplementary Figure 1b). The beach morphodynamic types[2] of the open-ocean beaches are modally low tide terrace or transverse bar and rip (Supplementary Table 1), whereas the embayed ones are mostly reflective in the upper beachface with sand flats lower in the intertidal zone. Our beach type distribution corresponds with the overall beach type occurrences typically found in the 1,230 km long Victorian coastline[3].

Suppl. Table 1
Beaches summary information.

Location	Environment	Beach Type	Orientation	Median Off. Sig. Wave Height (m)	Beach Length (m)	Surveyed (m)
Port Fairy	open-ocean	LTT / TBR	SE	2.87	5,800	2,120
Warrnambool	open-ocean	LTT / TBR	E - SE	2.87	3,500	2,023
Marengo	open-ocean	LTT / TBR	E	1.97	1,800	870
Apollo Bay	open-ocean	LTT / TBR	SE	1.97	3,000	1,947
Pt. Roadknight	open-ocean	LTT	E	1.97	700	751
St. Leonards	embayed	R + sf	E	0.89	3,000	2,697
Portarlington	embayed	R + sf	N	0.89	2,500	649
Cowes	embayed	R + sf & tc	N	na	5,750	2,235
Inverloch	open-ocean	R + ts	S	1.87	1,800	1,457
Seaspray	open-ocean	RBB / LBT	SE	1.31	2,000	2,337

LTT: low tide terrace; TBR: transverse bar and rip; RBB: rithmic bar and beach; LBT: longshore bar and through;
sf: sand flats; tc: tidal channels; ts: tidal shoals.
Median off. sig. wave height: median offshore significant wave height from 01.01.1985 to 19.06.2020 derived from multiple satellite radar altimeters.

The open-ocean locations have limited seasonality of storm-track positions and intensities, wind speed and swell height compared to the northern hemisphere, resulting in year-round west to east storms [4–6]. Moreover, the Southern Ocean has experienced the highest increase in mean wind speed and significant wave height extreme conditions (90th percentiles) in the world during the last 30 years[7] and it is projected to increase in the future[8].

Supplementary Method "Citizen scientists, UAV surveys and photogrammetric details"

For this work, citizen scientists are defined as members of the public who volunteer in the acquisition of aerial image data and ground control points for photogrammetric purposes. They have been invited to volunteer through social and traditional media channels or directly approaching local land managers and established community groups. Citizen scientists in our project can have two main roles: (1) Unmanned Aerial Vehicles (UAV) pilots, who are in charge of the UAV flight operations and (2) survey assistants, who assist in the Ground Control Points (GCP) survey while also acting as hazard spotters (e.g. air and pedestrian traffic). As a result, citizen scientists range from primary-school students to retirees.

For this work, each group received:

- 10 PropellerAero AeroPoints (*Propeller Aerobotics Pty Ltd,* https://www.propelleraero.com/) GCPs
- two AeroPoint bags
- one DJI Phantom 4 Pro/Advance (*SZ DJI Technology Co.,*https://www.dji.com/au) UAV
- one DJI Phantom 4 Pro/Advance transmitter
- one DJI USB to Micro USB adapter On The Go cable
- two DJI Phantom 4 Pro/Advance gimbal chocks
- three DJI Phantom 4 Pro/Advance batteries
- one DJI Phantom 4 Pro/Advance battery and transmitter charger
- one 3-in-1 DJI Phantom 4 Pro/Advance battery charging board
- eight DJI Phantom 4 Pro/Advance propellers
- one Apple iPad mini (*Apple Inc.,*https://www.apple.com)
- one Lightning to USB cable
- one Launch mat
- one UAV manual

The imaging system consists of a 12.833 x 8.556 mm CMOS sensor acquiring 20 megapixel RGB images of 5472 x 3648 pixel size (3:2 aspect ratio) with a rolling shutter system.

The UAV autonomous acquisition missions are planned with Pix4DCapture software (*Pix4D SA*, https://www.pix4d.com/product/pix4dcapture). Flights are performed at a nominal height of 100 m from Australian Height Datum (AHD) and nominal speed of 10 m/s, following a double-grids (cross-hatch) strategy with 70% of both front and side overlap, obtaining a ground sampling distance of approximately 0.025 m. The sensor is oriented at near-nadir (80°) to the ground. UAV surveys are performed approximately every 6 weeks, when wind speed (and gusts) do not exceed 15 knots (28 km/h), air temperature is below 35° C and it is free of rain. The targeted survey time is at low tide. Weather conditions and volunteers availability also affects the time of survey.

Each group also performs the GCP survey at each flight, using the 10 PropellerAero AeroPoints. The AeroPoints are sturdy smart 0.5 x 0.5 m targets with an inbuilt high-precision post-processed kinematic global navigation satellite system receiver that offer positional accuracies of 20 mm in x and y and 50 mm in z coordinates (post-processed). The spatial distribution of GCPs is evenly distributed alongshore, clear from trees and power lines and covering intertidal and foredune elevation levels alternatively. GCPs are left recording their position continuously for at least 45 minutes or for the whole duration of the flights, to a maximum of 5 hours.

The aerial images and GCP data collected by the citizen scientists are then processed by Deakin University and the University of Melbourne professional researchers using the photogrammetric software Pix4Dmapper V4.3.31 (*Pix4D SA*, https://www.pix4d.com/). A perspective lens camera model is used to estimate three radial and two tangential distortion parameters within the bundle-block adjustment procedure, which in average returned a satisfactory 0.49 % relative difference between initial and optimised internal camera parameters. Less than 1 % of the images are usually discarded due to poor calibration.

During the matching procedure, the median number of keypoints per calibrated image is on average 41,267.04, whereas an average of 7,656 matches are found per calibrated images. All images are

processed at full scale during the keypoints detection and matching process. During the densification process, half-scaled images are used. Overall, an average density of 168.7 points per m^3 has been obtained throughout densified point clouds, across surveys.

Prior to the DSM creation, the densified point clouds are pre-processed with noise filtering and surface smoothing (sharp type) algorithms. Then, the inverse distance weighting interpolation method has been used to create continuous raster surfaces of 0.025 m pixel size. The three-dimensional positions of the GCPs are used to georeference the dataset. On average, we obtained a georeferencing root mean squared error of 0.015 m.

Supplementary Method "Independent checkpoint surveys"

Two independent checkpoint surveys were conducted on 29th November 2018 (Supplementary Figure 2) and 11th December 2019 (Supplementary Figure 3) in Warrnambool to independently evaluate the vertical accuracy of the digital surface models generated by the citizen science protocol. The 2018 UAV survey was completed in 6 flights, each covering approximately 600 m of alongshore beach (300 m of radius from the UAV pilot), from 11:05 am (low tide at 10:37 am) to 12:10 am (tidal height change less than 0.1 m), with good and stable lightning conditions, using 9-10 smart ground control points per flight (total of 55). The flights were performed by 6 groups of 3 - 4 undergraduate students (total of 21) from Deakin University. The 2019 UAV survey was performed in 3 flights, from 9:27 am (8:17 am low tide) to 11:01 am (tidal height change less than 0.1 m) with stable lightning conditions. During the 2019 survey, a technical failure of the ground control points normally used in the protocol led to the manual georeferencing of the 3D scene using permanent features across the scene. Despite this survey not being included in the volumetric or beach dynamic analysis and no technical failures occurred during the surveys used for this study, this situation represents an opportunity to compare the vertical accuracies of the citizen science protocol during worst-case (2019) and operational (2018) scenarios.

During the two surveys, a total of 523 checkpoints were surveyed with a centimetre-accuracy Real-Time Kinematic Global Positioning System (RTK-GPS) following two different sampling schemes.

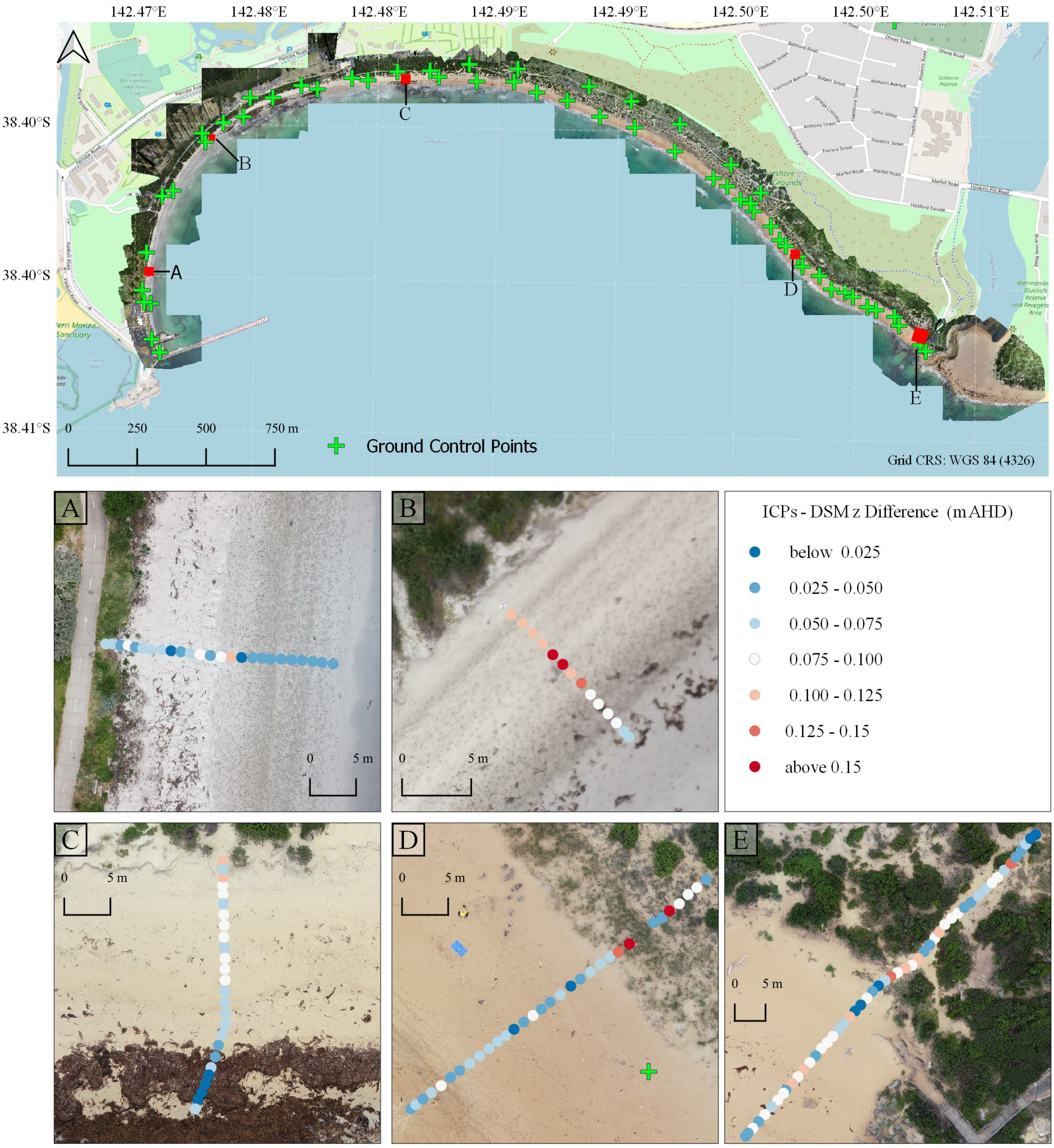

Suppl. Fig. 2. Map of error distribution of 2018 independent checkpoint (ICP) validation dataset shows overall vertical precision is in the 0.05 - 0.075 m range or better, increasing going further from the foredune vegetation. The thematic basemap has been sourced from OpenStreetMap contributors at https://www.openstreetmap.org/copyright. UAV

data collected the 29th November 2018 following our protocol and accessible at https://www.propelleraero.com/ (email: vcmp@deakin.edu.au; password: propellervcmp).

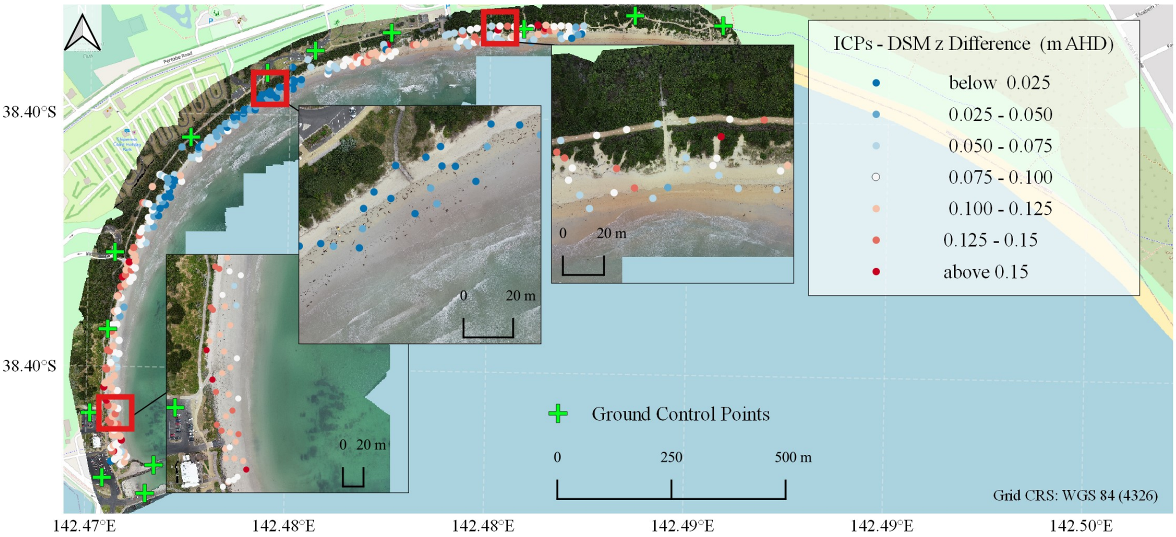

Suppl. Fig. 3. Map of worst case scenario error distribution of 2019 ICP validation dataset. The overall vertical precision is higher in the central part of the beach and generally increases going seaward cross-shore from the foredune, especially in the central and lower western side of the beach. The thematic basemap has been sourced from OpenStreetMap contributors at https://www.openstreetmap.org/copyright. UAV data collected the 11th December 2019 following our protocol and accessible at https://www.propelleraero.com/ (email: vcmp@deakin.edu.au; password: propellervcmp).

In the 2018 independent checkpoint survey, 208 checkpoints were collected along 7 cross-shore transects covering the backdune, foredune, incipient dune, intertidal beach and swash, while in the 2019 independent checkpoint survey, 315 points were collected randomly across the beachface, foredune and part of the backdune. When estimating error as the vertical difference from checkpoints and collinear digital surface models values, low quality RTK-GPS points have been discarded, finally resulting in 150 and 314 valid checkpoints for the 2018 and 2019 surveys respectively.

During the 2019 aerial survey a technical failure of the smart ground control points led to the manual georeferencing of the 3D scene using well-known permanent features across the scene.

The choice of the error metric depends on the error statistical distribution. Despite the Root Mean Squared Error (rmse) being the most used error metric in the literature[9], its validity is robust only when a normal distribution of absolute errors with no outliers is assumed, which is seldom occurring due to filtering and interpolation errors introduced by the digital photogrammetric procedure[10,11]. The normalised median absolute deviation (nmad) is reported to be a more robust estimator for elevation precision of photogrammetric products, in case the above mentioned assumptions are not met[10,12,13]. Accordingly, in addition to performing statistical tests (Shapiro-Whilk and D'Agostino-Pearson tests), we evaluated the normality of the absolute error distribution by visually assessing their Q-Q plots, as recommended in D'Agostino et al. (1990) and Höhle and Höhle (2009).

Supplementary Discussion “Limit of detection analysis”

Limits of Detections (LoD) incorporate intrinsic (structure from motion with multi-view stereo procedure, camera radial distortion, ground control points accuracy, interpolation and filtering artifacts) and extrinsic (surface texture, illumination, topographic effects) errors that propagated throughout the data acquisition and processing phases. In this study, we use LoD analysis (1) to compute reliable volume changes and (2) as a proxy for digital surface models errors used to investigate the inter-group overall end-products data quality differences. In Supplementary Figure 4a an example of LoD derivation and error normality evaluation in Apollo Bay is shown.

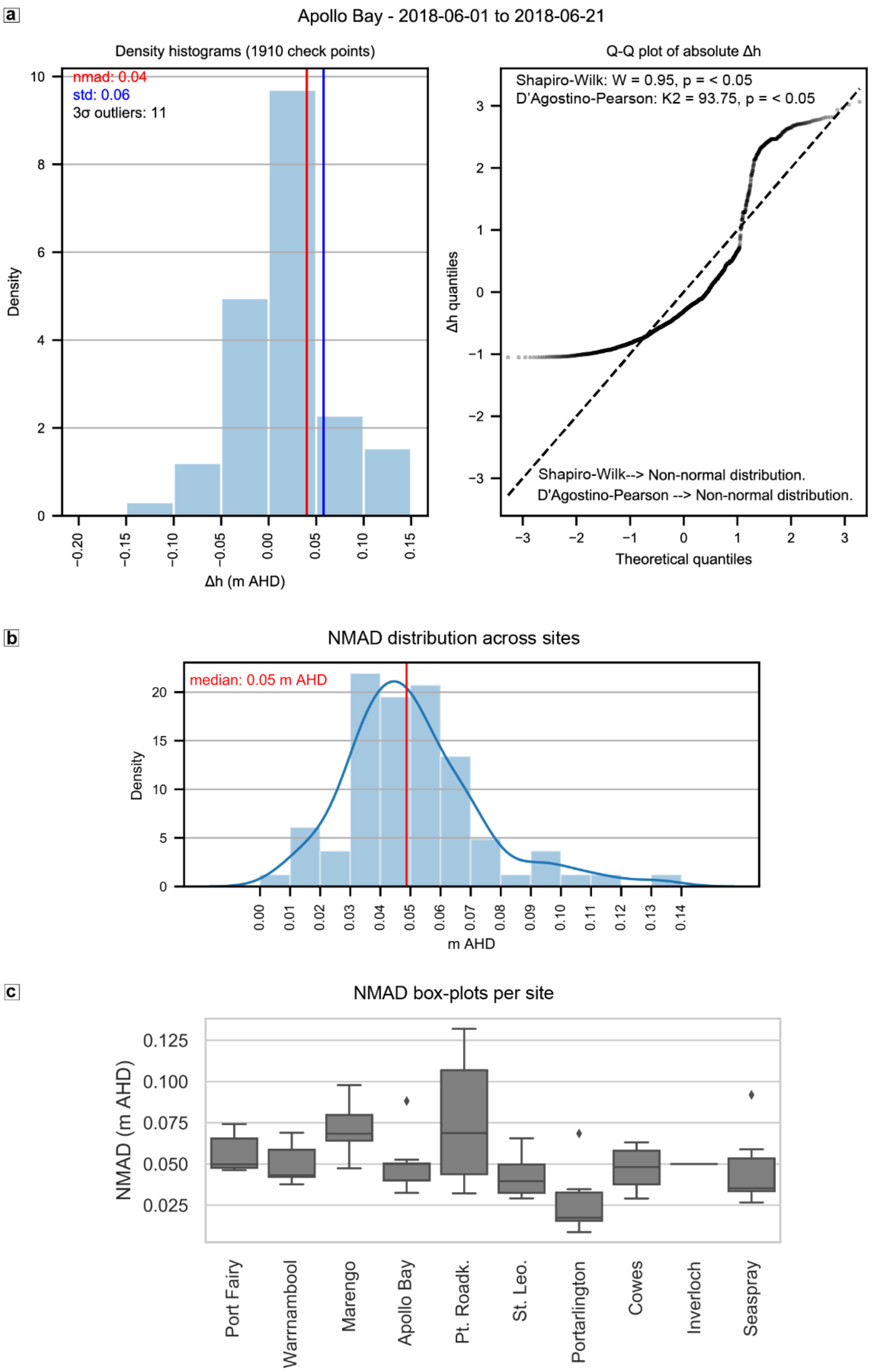

Suppl. Fig. 4. The procedure to compute the limit of detections employed in this research. (a) On the left, the distribution of absolute elevation change (Δh) of 1,910 checkpoints plotted along pseudo-invariant features in the first Apollo Bay time period, with normalised median absolute deviation (nmad) in red and standard deviation (std), in blue. Note how 11 values beyond 3 times the standard deviation (3σ) have been excluded from the plot for visualisation purposes.

On the right, the Q-Q plot used in combination with multiple statistical tests are used to evaluate the normality of each distribution and decide the best error metric (estimator) for the LoD. (b) The global nmad distribution indicates a very satisfactory median value of 0.05 m (n=73). (c) Site-specific nmad distribution with the medians displayed as the inner horizontal line, used for the Kruskal-Wallis test. Note how in Inverloch, due to the lack of pseudo-persistent features near the beach, the global median LoD of 0.05 m has been used.

The Q-Q plots and statistical tests of absolute elevation difference (Δh) indicated a non-normal distribution in all cases, resulting in the nmad to be chosen as the error metric to obtain the LoDs. Overall, a median LoD value of 0.05 m (n=73) has been computed (Supplementary Figure 4b). At the location level (Supplementary Figure 4c), despite Point Roadknight range being considerably higher than the others, all the sites show compact LoDs distributions, reflecting consistency and stability in the inter-group citizen science protocols. The Kruskal-Wallis H test demonstrated that no significant differences ($H = 16.167$, $p = 0.063$, n=73) exist between each location specific LoD distributions at the 0.05 significance level. This means that the error distributions of citizen scientists' digital surface models originate from the same general distribution.

Supplementary Discussion "Citizen scientists' accuracy"

To validate the vertical accuracy of citizen scientists' digital surface models in an operational scenario we used the independent checkpoint method, proved the non-normality and presence of outliers in the absolute error distribution and obtained an nmad of 0.048 m and an rmse of 0.089 m.

In the UAV-SfM beach monitoring literature, although other methods and metrics have been used, the great majority of researchers evaluated vertical accuracy with independent checkpoints and root mean squared error (rmse), with only two studies using robust statistics due to prior error distribution evaluation (Supplementary Table 2). For this reason, in addition to the more appropriate and robust normalised median absolute deviation (nmad), we also compared our rmse value with those reported in the literature (Supplementary Figure 5).

Suppl. Table 2
UAV-SfM vertical accuracies reported in the beach erosion literature

Reference	Flight Height (m)	Vertical Accuracy (m)	Error Metric	Method
Our study	100	0.048	nmad	icp
Our study	100	0.089	rmse	icp
Casella et al. 2020 [41]	30-50	0.05	rmse	icp
Laporte-Fauret et al. 2020 [15]	65	0.046	rmse	icp
Rotnicka et al. 2020 [16]	350	0.074	rmse	icp
Zanutta et al. 2020 [17]	53.1 - 113	0.02 - 0.09	rmse	icp
Gómez-Pazo et al. 2019 [18]	30	0.04	std	lod
Jayson-Quashigah et al. 2019 [19]	150	0.009 - 0.2	rmse	propagated error
Kim et al. 2019 [20]	100	0.04	rmse	tls
Laporte-Fauret et al. 2019 [21]	65	0.05	rmse	icp
Pagán et al. 2019 [22]	60	0.173	rmse	icp
Chen et al. 2018 [23]	150	0.11	rmse	icp
Gonçalves et al. 2018a [24]	80-100	0.06	nmad	icp
Gonçalves et al. 2018b [13]	50-170	0.06-0.05	rmse	icp
Guisado-Pintado et al. 2018 [25]	100	0.021	rmse	propagated error
Ierodiaconou et al. 2018 [26]	100	0.04	std	lod
Ruessink et al. 2018 [27]	na	0.067 - 0.107	sd	icp
King et al. 2017 [36]	70	0.068	rmse	icp
Pádua et al. 2017 [28]	na	0.046 - 0.071	rmse	icp
Brunier et al. 2016 [12]	280	0.07	nmad	icp
Casella et al. 2016 [37]	70	0.15-0.16	rmse	icp
Long et al. 2016a [38]	149	0.091	rmse	icp
Long et al. 2016b [29]	150	0.16-0.17	rmse	icp
Papakonstantinou et al. 2016 [30]	100	0.004 - 0.028	rmse	gcp
Turner et al. 2016 [31]	100	0.026	rmse	UAV-ATV
Gonçalves and Henriques 2015 [32]	131-137	0.027 - 0.046	rmse	icp
Harwin et al. 2015 [39]	18	0.005 - 0.02	rmse	icp
Casella et al. 2014 [33]	80	0.12	rmse	icp
Mancini et al. 2013 [34]	40	0.11	rmse	icp
Harwin and Lucieer 2012 [40]	30-50	0.04	rmse	icp

icp: Indipendent Check Points; lod: Limit of Detection in calibration zones; gcp: non-indipendent ground control points; propagated error: see Wheaton et al. 2009 [35]; UAV-ATV: UAV model compared with all-terrain vehicle RTK-GPS survey.

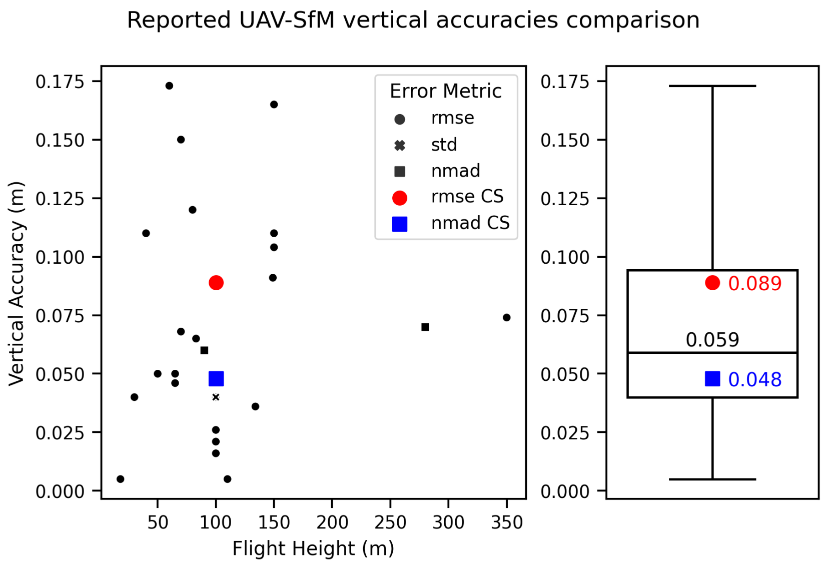

Suppl. Fig. 5. Comparison of the vertical error of the citizen science protocol assessed with ICPs with similar studies reported in the scientific literature. (a): vertical errors over nominal flight heights reported in root mean square error (rmse), standard deviation (std) and normalised median absolute deviation (nmad), including all error assessment methods; (b) box-plot of reported vertical errors showing their median value (0.059 m) and citizen science rmse (0.089 m) and nmad (0.048 m) values.

If we consider rmse (0.089 m), citizen scientists working under our protocol provide data approximately 3 cm less accurate of the median rmse (0.059 m, n=28, Supplementary Figure 5b) reported by professional researchers across relevant scientific literature (Supplementary Figure 5a). However, these studies assumed or reported normal distribution of the checkpoint errors and no outliers, which is not our case and in general rarely occurs when dealing with photogrammetric datasets [10]. Therefore, using the more appropriate and robust nmad as error metrics, citizen scientists obtained the best vertical accuracy reported in the literature so far, although nmad has currently been used in only two other studies [12,14]. Given the aforementioned observations, we conclude that citizen scientists under our protocol can provide beach topographic data as accurate as professional researchers.

Supplementary Discussion "Role of legislation"

While all members of the public are able to participate in this study as survey assistants, those who wish to operate the UAVs must register with the Australia Civil Aviation Safety Authority (CASA). Thus, differently from other coastal citizen science projects where anyone can take part as a citizen scientist [15,16], in our approach, legislation plays a central role in defining who can be a pilot,.

In Australia, scientific operations with UAVs < 2 kg are part of the 'sub-2 kg excluded category", which allows individuals to fly small UAVs within standard operating conditions without the need of a remote pilot licence. CASA requires them to be older than 16 years, obtain an aviation reference number and to become accredited operators by passing a free online assessment. No formal risk assessment is required by CASA.

Nevertheless, citizen scientists employed in this work must complete an additional 2-days training program in the theory and practical operation of UAVs for photogrammetric surveys in sandy beaches, organised by Deakin University. This includes knowledge of the regulations, weather, documentation, record keeping, the safe set-up and pack down of the UAV and a flight competency test in both normal operating conditions and abnormal conditions (i.e. simulating a failure in the UAV GPS system). Deakin University requires them to complete flight authorisation and risk assessment

forms for each site prior to the beginning of the monitoring. Moreover, all groups working for this project are awarded public liability insurance by the Victorian Department of Environment, Land, Water and Planning.

In general, the key legislation requirements that need to be met in order to perform and replicate the UAV missions implemented by citizen scientists in this work are that (1) UAV flights are permitted over areas of interest, (2) automatic (waypoint-based) UAV flight mode is allowed and (3) no mandatory UAV flight license is required for research applications using sub-2 kg airframes. As UAV laws vary around the World, the harmonisation of different countries' directives into regional or even continental scale UAV regulations will greatly increase the applicability of our protocol and widen its scope. Promisingly, such a standardisation process has already begun in Europe.

The European Union Aviation Safety Agency (EASA) regulates civil aviation legislation and sets common standards for 28 countries of the European Union and 4 non-EU countries (Switzerland, Norway, Iceland and Liechtenstein). According to the published EASA timeline, from the 31st December 2020 UAV users can start operating in the “Open” category, which will allow citizen scientists (referred to drone operators in EASA legislation) older than 16 years of age to perform automatic UAVs operations, provided they register themselves and pass EASA online knowledge tests. The new EASA UAV legislation will resemble very closely to the Australian one, allowing UAV citizen science projects to take place in 32 EASA countries and potentially enabling beach monitoring in 25 EASA coastal countries. However, a greatest number of coastal no-fly zones (due to controlled aerospace from the military, proximity to aerodromes or natural protected areas) in combination with crowded beaches can limit beach monitoring in EASA countries.

As for small island developing countries, national civil aviation authorities' UAV regulations range from total ban (Cuba and Barbados) to “Australia-like” approach (Papua New Guinea), with important variants that could limit the feasibility or replicability of our protocol. For instance, in Maldives, Dominica, Guyana and Jamaica, research use of sub-2kg UAVs is subject to the approval of multiple local authorities and flying permits must be obtained, without any specific license requirements, but with the caveat that a formal licence increases the likelihood of obtaining the permit. This could reduce the potential number of members of the community who can pilot the

UAVs and direct liaison with local authorities and special approvals might be needed, impacting the feasibility of UAV citizen science. Moreover, in the Dominican Republic, automatic flight modes are banned. Manual flights not only introduce unwanted pilot-specific bias but possibly cause temporal inconsistencies of flight parameters (frontal and lateral image overlaps, flight altitude, speed of flight, ground sampling distance) which impact the photogrammetric reconstruction, finally reducing replicability. Thus, while in EASA countries our citizen science protocol could be realizable (with limitations), in insular developing countries its implementation is currently challenged by total bans, stricter or less defined UAV regulations.

Supplementary Discussion "Monetary costs"

The initial cost of setting up a group in one location for the first year is US$25,000, which includes the UAV surveying set, online data processing and hosting and the time to train and support a group of 3-4 citizen scientists.

However, yearly monitoring running costs once a group is established are approximately US$10,740 per location. In total, 10 UAVs (one each group/location) and 100 smart ground control points have been used in this first year study, totalling a cost of US$250,590 or US$3,020 per survey (total area of 40 km^2). In the following years, the cost will be US$107,390 or US$1,294 per survey.

The cost effectiveness of this approach is tied to the data spatiotemporal resolution rather than its cost per square kilometre. Potentially, once established, groups could survey sites more frequently or for an extended period of time further reducing the cost per survey.

Supplementary Discussion "Apollo Bay behavioural regime sensitivity analysis"

For a reliable computation of behavioural regime (r-BCD index) at the transect scale the elevation difference (Δh) points within the subaerial beachface must be beyond the period-specific limit of

detections. This filtering process assures that only high quality data is used in the r-BCDs computation to the detriment of the total number of valid observations per transect. Moreover, two additional filtering steps are applied at the site-level, to ensure that are only retained (1) points that remain valid for at least a certain amount of time periods (*t*) and (2) transects that have a number of valid points greater than a determined minimum threshold (*pt*, Supplementary Figure 6). This is done to ensure comparability across time and transects in a determined location. Therefore, *t* is an important parameter that should ideally be as high as the available time periods, in order to ensure that the maximum behavioural variability is captured. Yet, setting this parameter very high can reduce considerably the number of valid points retained in a single transect, which in turn can fall below *t*, leading to the loss of transects from the final behavioural map. It is also informative to monitor and try to minimise the number of transects that passed from a depositional to erosional behavioural regime (or vice versa) in the last time period (i. e. changed sign from *t*-1 to *t*), for any chosen *t*. Those transects could signal a behaviour that only emerged by choosing a determined value for *t*, signalling a potentially lower confidence in their r-BCD values. For these reasons, we performed a sensitivity analysis to choose a sub-optimal combination of *t* and *p* such as (1) at least 85% of the total transects are retained and (2) a reasonably low number of sign changes occurred (Supplementary Figure 6).

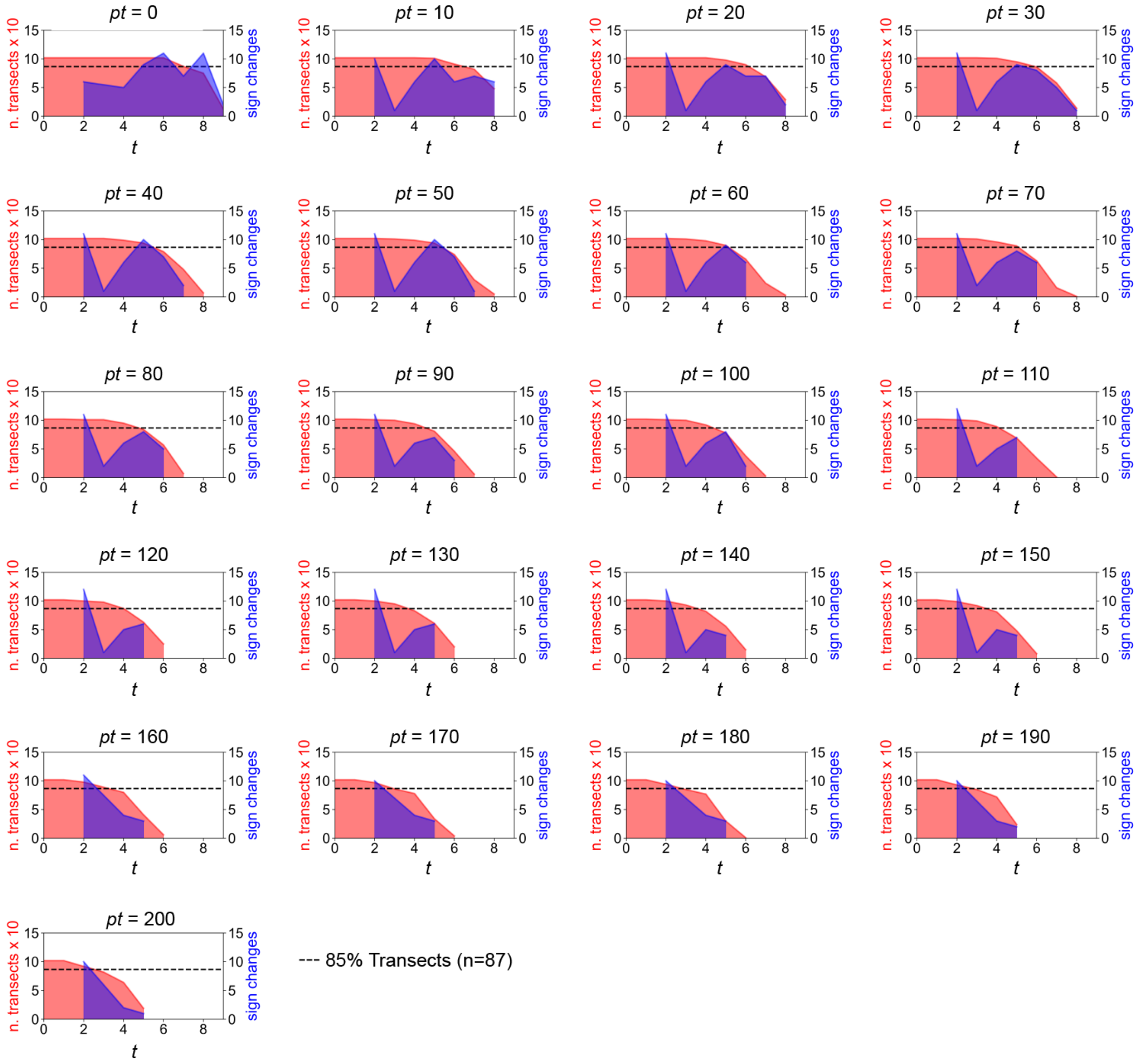

Suppl. Fig. 6. Sensitivity analysis for r-BCD computation in Apollo bay. Plots showing the total number of retained transects (in red) and number of transects that changed sign (in blue) as a function of the minimum number of times a transect must be valid to be considered in the analysis (*t*), for different minimum points thresholds (*pt*). Logically, a higher number of *pt* will reduce the chances of having enough valid observations per transects that remain valid for any given *t*, reducing the number of retained transects *t*. As such, we chose *pt*=10 and *t*=7 as these parameters ensure that 85% of the total available transects are retained with a reasonably low number of sign changes (7) (signalled with a dot in Figure 4).

In Apollo Bay, a total of 9 periods are analysed. Retaining points that were valid throughout the totality of the timeseries (*t*=9) and no minimum points threshold (*pt*=0) results in only 13 transects (out of 102) retained, whereas 0 transects for any *pt* > 0.

On the other hand, with *t*=0 all 102 transects are retained with 0 < *pt* < 200.

In case we choose *t*=5, the number of retained transects range from a maximum of 102 with 7 sign changes (*pt*=0) to a minimum of 22 with 3 sign changes (*pt*=200).

Therefore, by visually inspecting Supplementary Figure 6, the sub-optimal parameters chosen for Apollo Bay are (1) *pt*= 10 and (2) *t*= 7, resulting in 77 transects and 7 sign changes. The transects that changed sign occurred at 180 m, 840 m, 860 m, 1120 m, 1540 m, 1560 m and 1580 m alongshore and are signalled with a dot at their seaward base in Figure 4.

Supplementary Figures

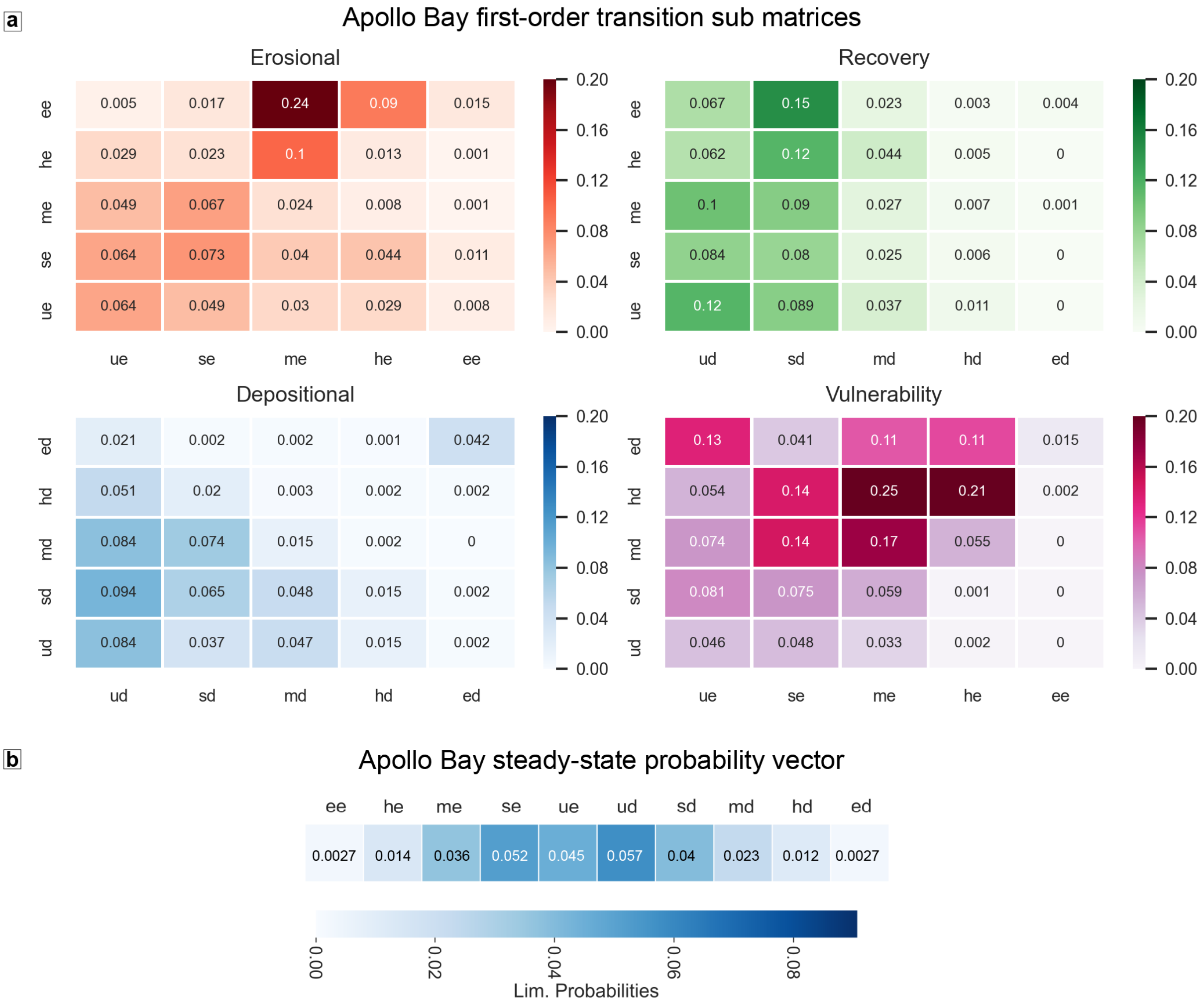

Suppl. Fig. 7. Apollo Bay visual representations of transition matrices and steady-state probability vector. (a) Re-arranged sub-matrices derived from the full transition matrix. The row labels are the "from" states while the column labels are the "to" states; (b) Site-level steady-state vector of Apollo Bay used to compute its residual beachface cluster dynamics index (behavioural regime). Note: the probabilities in both (a) and (b) do not sum to 1 as the significant hotspot to spatial outlier transition class has been not retained in the analysis as do not carry beach dynamic information.

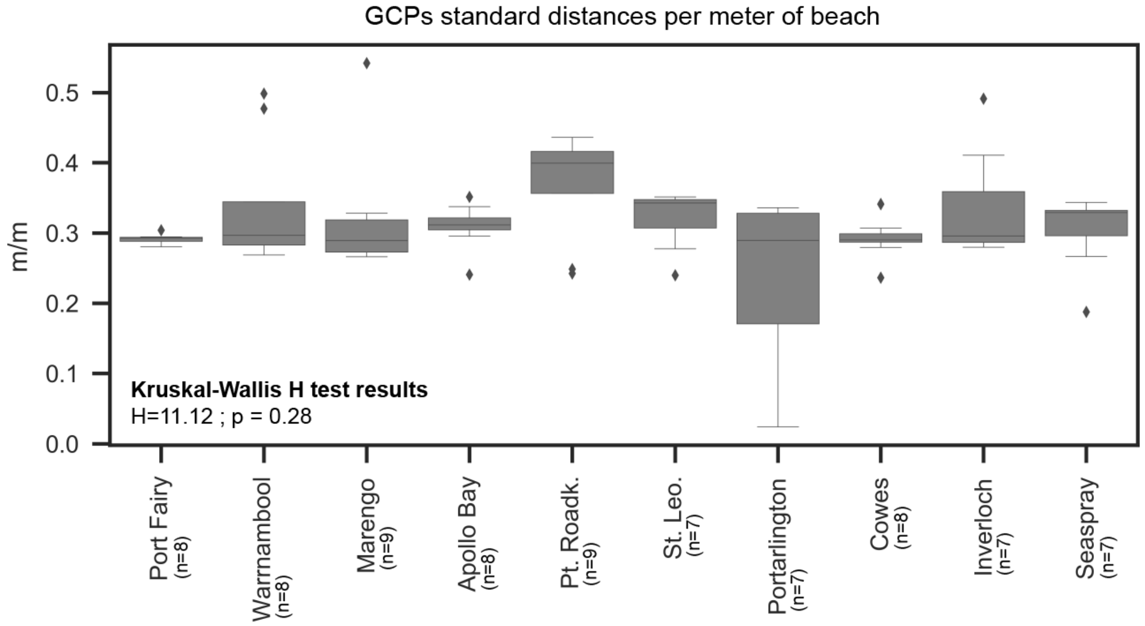

Suppl. Fig. 8. GCPs spatial dispersion across locations does not significantly change (p=0.005). As a proxy for ground control points (GCPs) spatial dispersion we computed the standard distance metric for each survey GCPs configuration, which returns the mean Euclidean distance of each GCP from the geometric centre of the whole GCPs set. To account for longer beaches, which can induce volunteers to increase the spread between GCPs, we normalised the standard distance with beach length, resulting in the m/m unit of measure.

Supplementary Tables

Suppl. Table 3

Error metrics of ICP surveys .

Survey	ME	STD	RMSE	MAE	NMAD
2018-11-29	-0.044	0.077	0.089	0.071	0.048
2019-12-11	0.128	0.063	0.143	0.130	0.054

ME: mean error; STD: standard deviation;
RMSE: root mean square error; MAE: mean absolute error;
NMAD: normalised median absolute deviation

Supplementary References

1. DWELP. *Victorian Coastal Hazard Assessment 2017 (No. 1)*. (The State of Victoria Department of Environment, Land, Water and Planning, 2017), at https://www.marineandcoasts.vic.gov.au/__data/assets/pdf_file/0021/122709/VCHA2017_R1_Victorian_Coastal_Hazard_Assessment_2017_Final_R1.compressed.pdf
2. Wright, L. D. & Short, A. D. Morphodynamic variability of surf zones and beaches: A synthesis. *Mar. Geol.* **56**, 93–118 (1984).
3. Short, A. D. *Beaches of the Victorian Coast & Port Phillip Bay: A Guide to Their Nature, Characteristics, Surf and Safety*. (Sydney University Press, 1996).
4. Young, I. R. Seasonal variability of the global ocean wind and wave climate. *Int. J. Climatol.* **19**, 931–950 (1999).
5. Trenberth, K. E. Storm Tracks in the Southern Hemisphere. *J. Atmos. Sci.* **48**, 2159–2178 (1991).
6. Babanin, A. V. *et al.* Waves and Swells in High Wind and Extreme Fetches, Measurements in the Southern Ocean. *Frontiers in Marine Science* **6**, 361 (2019).
7. Young, I. R. & Ribal, A. Multiplatform evaluation of global trends in wind speed and wave height. *Science* **364**, 548–552 (2019).
8. Morim, J., Hemer, M., Cartwright, N., Strauss, D. & Andutta, F. On the concordance of 21st century wind-wave climate projections. *Glob. Planet. Change* **167**, 160–171 (2018).
9. Carrivick, J. L., Smith, M. W. & Quincey, D. J. *Structure from Motion in the Geosciences*. (John Wiley & Sons, 2016).
10. Höhle, J. & Höhle, M. Accuracy assessment of digital elevation models by means of robust statistical methods. *ISPRS J. Photogramm. Remote Sens.* **64**, 398–406 (2009).
11. Wang, B., Shi, W. & Liu, E. Robust methods for assessing the accuracy of linear interpolated DEM. *Int. J. Appl. Earth Obs. Geoinf.* **34**, 198–206 (2015).
12. Brunier, G., Fleury, J., Anthony, E. J., Gardel, A. & Dussouillez, P. Close-range airborne Structure-from-Motion Photogrammetry for high-resolution beach morphometric surveys: Examples from an embayed rotating beach. *Geomorphology* **261**, 76–88 (2016).

13. Gonçalves, J. A., Bastos, L., Madeira, S., Magalhães, A. & Bio, A. Three-dimensional data collection for coastal management – efficiency and applicability of terrestrial and airborne methods. *Int. J. Remote Sens.* **39**, 9380–9399 (2018).

14. Gonçalves, G. R., Pérez, J. A. & Duarte, J. Accuracy and effectiveness of low cost UASs and open source photogrammetric software for foredunes mapping. *Int. J. Remote Sens.* **39**, 5059–5077 (2018).

15. Hart, J. & Blenkinsopp, C. Using Citizen Science to Collect Coastal Monitoring Data. *coas* **95**, 824–828 (2020).

16. Harley, M. D., Kinsela, M. A., Sánchez-García, E. & Vos, K. Shoreline change mapping using crowd-sourced smartphone images. *Coast. Eng.* **150**, 175–189 (2019).

2. Wright, L. D. & Short, A. D. Morphodynamic variability of surf zones and beaches: A synthesis. *Mar. Geol.* **56**, 93–118 (1984).

15.Laporte-Fauret, Q. *et al.* Coastal Dune Morphology Evolution Combining Lidar and UAV Surveys, Truc Vert beach 2011-2019. *J. Coast. Res.* **95**, 163 (2020)

16.Rotnicka, J. *et al.* Accuracy of the UAV-Based DEM of Beach–Foredune Topography in Relation to Selected Morphometric Variables, Land Cover, and Multitemporal Sediment Budget. *Estuaries Coasts* (2020)

17.Zanutta, A., Lambertini, A. & Vittuari, L. UAV Photogrammetry and Ground Surveys as a Mapping Tool for Quickly Monitoring Shoreline and Beach Changes. *J. Mar. Sci. Eng.* **8**, (2020)

18.Gómez-Pazo, A., Pérez-Alberti, A. & Trenhaile, A. Recording inter-annual changes on a boulder beach in Galicia, NW Spain using an unmanned aerial vehicle. *Earth Surf. Processes Landforms* **44**, 1004–1014 (2019)

19.Jayson-Quashigah, P.-N., Addo, K. A., Arnisigo, B. & Wiafe, G. Assessment of short-term beach sediment change in the Volta Delta coast in Ghana using data from Unmanned Aerial Vehicles (Drone). *Ocean Coast. Manag.* **182**, (2019)

20.Kim, S. *et al.* Feasibility of UAV Photogrammetry for Coastal Monitoring: A Case Study in Imlang Beach, South Korea. *J. Coast. Res.* 386–392 (2019)

21.Laporte-Fauret, Q. *et al.* Low-Cost UAV for High-Resolution and Large-Scale Coastal Dune Change Monitoring Using Photogrammetry. *J. Mar. Sci. Eng.* **7**, 63 (2019)

22.Pagán, J. I., Bañón, L., López, I., Bañón, C. & Aragonés, L. Monitoring the dune-beach system of Guardamar del Segura (Spain) using UAV, SfM and GIS techniques. *Sci. Total Environ.* **687**, 1034–1045 (2019)

23.Chen, B. *et al.* High-resolution monitoring of beach topography and its change using unmanned aerial vehicle imagery. *Ocean Coast. Manag.* **160**, 103–116 (2018)

24.Gonçalves, G. R., Pérez, J. A. & Duarte, J. Accuracy and effectiveness of low cost UASs and open source photogrammetric software for foredunes mapping. *Int. J. Remote Sens.* **39**, 5059–5077 (2018)

25.Guisado-Pintado, E., Jackson, D. W. T. & Rogers, D. 3D mapping efficacy of a drone and terrestrial laser scanner over a temperate beach-dune zone. *Geomorphology* **328**, 157–172 (2019)

26.Ierodiaconou, D., Schimel, A. C. G. & Kennedy, D. M. A new perspective of storm bite on sandy beaches using Unmanned Aerial Vehicles. *Z. Geomorphol. Suppl.* **60**, 123–137 (2016)

27.Ruessink, B. G., Arens, S. M., Kuipers, M. & Donker, J. J. A. Coastal dune dynamics in response to excavated foredune notches. *Aeolian Research* **31**, 3–17 (2018)

28.Pádua, L. *et al.* Multi-Temporal Analysis of Forestry and Coastal Environments Using UASs. *Remote Sens.* **10**, 24 (2017)

29.Long, N., Millescamps, B., Guillot, B., Pouget, F. & Bertin, X. Monitoring the Topography of a Dynamic Tidal Inlet Using UAV Imagery. *Remote Sensing* **8**, 387 (2016)

30.Papakonstantinou, A., Topouzelis, K. & Pavlogeorgatos, G. Coastline Zones Identification

and 3D Coastal Mapping Using UAV Spatial Data. *IJGI* **5**, 75 (2016)

31.Turner, I. L., Harley, M. D. & Drummond, C. D. UAVs for coastal surveying. *Coast. Eng.* **114**, 19–24 (2016)

32.Gonçalves, J. A. & Henriques, R. UAV photogrammetry for topographic monitoring of coastal areas. *ISPRS J. Photogramm. Remote Sens.* **104**, 101–111 (2015)

33.Casella, E. *et al.* Study of wave runup using numerical models and low-altitude aerial photogrammetry: A tool for coastal management. *Estuar. Coast. Shelf Sci.* **149**, 160–167 (2014)

34.Mancini, F. *et al.* Using Unmanned Aerial Vehicles (UAV) for High-Resolution Reconstruction of Topography: The Structure from Motion Approach on Coastal Environments. *Remote Sens.* **5**, 6880–6898 (2013)

35.Wheaton, J. M., Brasington, J., Darby, S. E. & Sear, D. A. Accounting for uncertainty in DEMs from repeat topographic surveys: improved sediment budgets. *Earth Surf. Processes Landforms,* **25**, (2009)

36. King, S. *et al.* Condition survey of coastal structures using UAV and photogrammetry. *Australasian Coasts & Ports 2017: Working with Nature* **704** (2017)

37. Casella, E. *et al.* Drones as tools for monitoring beach topography changes in the Ligurian Sea (NW Mediterranean). *Geo-Mar. Lett.* **36**, 151–163 (2016)

38. Long, N. *et al.* ACCURACY ASSESSMENT OF COASTAL TOPOGRAPHY DERIVED FROM UAV IMAGES. *Int. Arch. Photogramm. Remote Sens. Spatial Inf. Sci.* **XLI-B1**, 1127–1134 (2016)

39. Harwin, S., Lucieer, A. & Osborn, J. The Impact of the Calibration Method on the Accuracy of Point Clouds Derived Using Unmanned Aerial Vehicle Multi-View Stereopsis. *Remote Sensing* **7**, 11933–11953 (2015)

40. Harwin, S. & Lucieer, A. Assessing the Accuracy of Georeferenced Point Clouds Produced via Multi-View Stereopsis from Unmanned Aerial Vehicle (UAV) Imagery. *Remote Sensing* **4**, 1573–1599 (2012)

41. Casella, E., Drechsel, J., Winter, C., Benninghoff, M. & Rovere, A. Accuracy of sand beach topography surveying by drones and photogrammetry. *Geo-Mar. Lett.* **40**, 255–268 (2020)
